# Synthesis of a benzoxazinthione derivative of tanaproget and pharmacological evaluation for PET imaging of PR expression

**DOI:** 10.1186/s41181-018-0054-z

**Published:** 2019-01-10

**Authors:** Louis Allott, Cecilia Miranda, Angela Hayes, Florence Raynaud, Christopher Cawthorne, Graham Smith

**Affiliations:** 1 Division of Radiotherapy and Imaging, The Institute of Cancer Research, 123 Old Brompton Road, London, UK; 2 PET Research Centre, University of Hull, Cottingham Road, Hull, Yorkshire HU6 7RX UK; 3 Cancer Therapeutics, The Institute of Cancer Research, 123 Old Brompton Road, London, UK; 4 Nuclear Medicine and Molecular Imaging, Department of Imaging and Pathology/MoSAIC- Molecular Small Animal Imaging Centre, Katholieke Universiteit Leuven, Leuven, Belgium

**Keywords:** PET, Fluorine-18, Progesterone, Steroid hormone receptor, Tanaproget, Fluoropyridine metabolism

## Abstract

**Background:**

The histological evaluation of estrogen receptor (ER) and progesterone receptor (PR) expression in breast cancer lesions from biopsy tissue can stratify patients to receive endocrine therapy. Furthermore, PR expression can predict response to selective estrogen receptor modulators (SERMs). Current immunohistochemical approaches to PR detection are limited by sampling error associated with biopsy and lack of standardised protocols; positron emission tomography (PET) using receptor targeted radiopharmaceuticals to provide quantitative, whole-body imaging may overcome these limitations. PR expression has been successfully imaged with PET in the clinical setting, however investigation into new radioligands with improved pharmacokinetics and metabolic stability is desirable.

**Results:**

We report the synthesis of a focused library of non-steroidal PR ligands evaluated for use as PET radioligands. A lead candidate (**[**^**18**^**F]2**) with low nanomolar activity was selected and radiolabelled with a radiochemical yield of 2.29 ± 2.31% (decay-corrected), radiochemical purity (RCP) > 95% and a molar activity of 2.5 ± 1.6 GBq/μmol. Cell uptake studies showed a significant and specific accumulation of **[**^**18**^**F]2** in T47D (PR++) breast cancer cell compared to MDA-MB-231 (PR-) control; however, in vivo evaluation was confounded by rapid defluorination of the radioligand. In vitro metabolite analysis of 2 in MLM confirmed defluorination and oxidative metabolism of the thiocarbamate to oxocarbamate moiety by mass spectrometry.

**Conclusions:**

A route to access **[**^**18**^**F]2** was developed to allow in vitro and in vivo evaluation, albeit with low radiochemical yield and modest molar activity. **[**^**18**^**F]2** demonstrated selective uptake in PR++ T47D cells which could be blocked in a dose dependent manner with progesterone. However, **[**^**18**^**F]2** showed poor in vivo metabolic stability with rapid defluorination within the time frame of the imaging protocol.

**Electronic supplementary material:**

The online version of this article (10.1186/s41181-018-0054-z) contains supplementary material, which is available to authorized users.

## Background

**Fig. 1:**
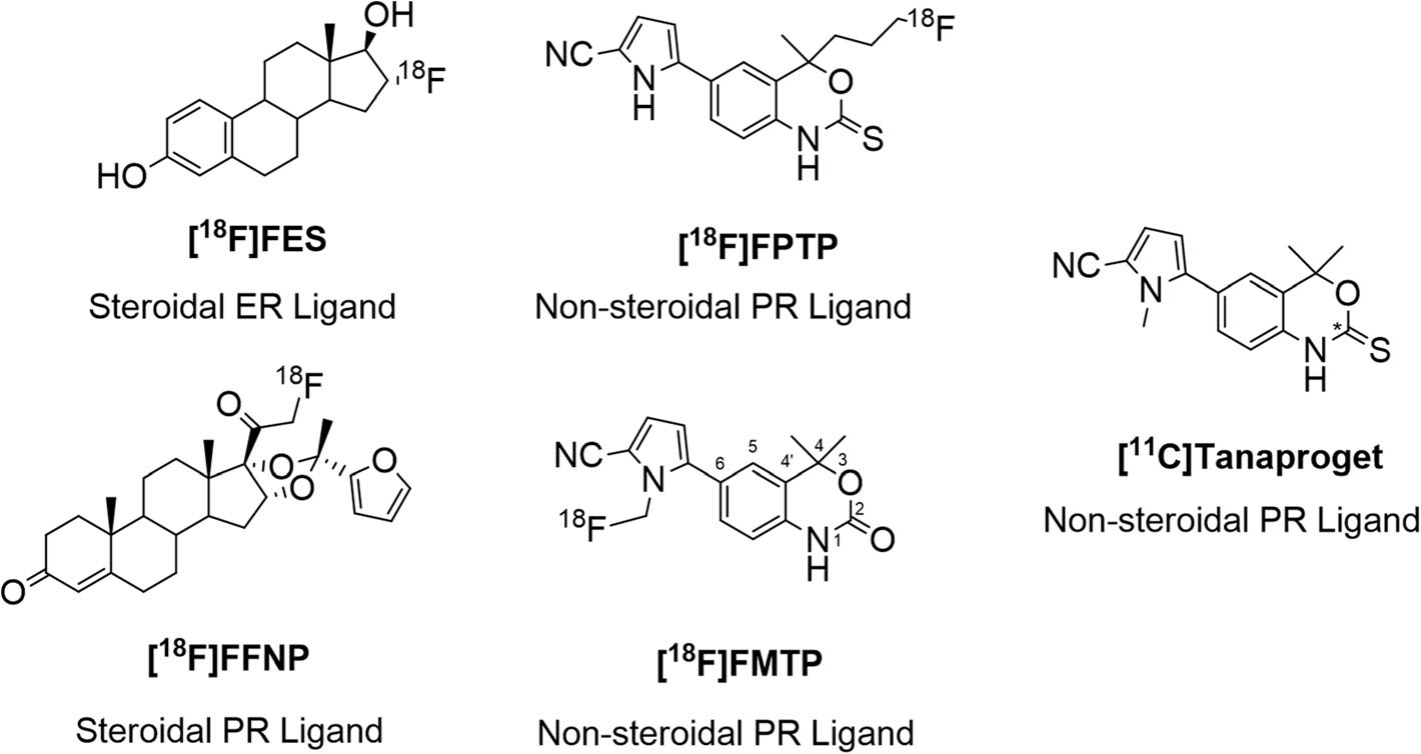
Structures of steroid hormone receptor radioligands: 16α-[^18^F]fluoro-17β-estradiol ([^18^F]FES), 21-[^18^F]fluorofuranyl-norprogesterone ([^18^F]FFNP), [^18^F]fluoropropyl-Tanaproget ([^18^F]FPTP), [^18^F]fluoromethyl-Tanaproget ([^18^F]FMTP) and [^11^C]Tanaproget. Substitutive nomenclature of benzoxazin(thi)one derivatives is demonstrated using [^18^F]FMTP

The steroid hormone receptor (SHR) family includes estrogen receptors (ERs), progesterone receptors (PRs) and androgen receptors (ARs) all of which have been studied using molecular imaging in cancer of the breast, endometrium and prostate (Mcguire, 1978, Suzuki et al., 2003). In most patients, ER-associated proliferative signalling is maintained by endogenous estradiol binding in the ER ligand-binding domain (LBD). Targeting ER with selective estrogen receptor modulators (SERMs) is a successful therapy in breast cancer for patients who express functioning ER (Osborne, 1998). The current standard of care affords the stratification of patients into potential responders/non-responders to endocrine therapy from histological evaluation of ER/PR expression in tissue from a primary lesion using a core-needle biopsy (Harvey et al., 1999, Osborne et al., 1980). The expression of PR can be used as a surrogate biomarker to report on ER function. As functional ER transcribes the PR gene, the upregulation of PR is predictive of a functioning estrogen-response pathway and therefore indicative of response to ER targeted endocrine therapy. Furthermore, treatment response can be monitored by comparing basal and post-treatment PR levels (Osborne et al., 1980). The poor representation of inter- and intra-tumour heterogeneity from single biopsies can lead to inaccurate assessment of ER status; furthermore, biopsy is not appropriate in the metastatic setting. As a consequence, a clinical dilemma can present where equivocal response from ER/PR status may result in inappropriate treatment planning. PET radiopharmaceuticals targeting SHRs exploit the minimally invasive, highly sensitive and quantitative, whole-body imaging modality to overcome the limitations of biopsy sampling. PET can be used as a predictive biomarker for patient stratification and/or a therapy response biomarker for monitoring treatment; in the case of breast cancer, both a predictive and therapy response marker are desirable (Banerji and Workman, 2016, Mankoff et al., 2008). Clinical trials to image ER expression with 16α-[^18^F]fluoro-17β-estradiol ([^18^F]FES) in primary and metastatic breast cancer were promising (Fig. 1) (Allott et al., 2015, Mintun et al., 1988). [^18^F]FES has been used as a predictive biomarker for stratifying patients to receive endocrine therapy (i.e. Tamoxifen™, Fulvestrant™) and as a therapy response biomarker to identify patients who are not responding to treatment. However, monitoring treatment response using [^18^F]FES, where the ligand binding domain (LBD) of ER is saturated by a therapeutic dose of endocrine therapy agent, requires sufficient time for drug washout for post-treatment scans. Therefore, [^18^F]FES is ideally suited for measuring pre-treatment target expression and drug occupancy of the receptor (Mankoff et al., 2017).

Imaging PR could potentially allow the monitoring of treatment response during endocrine therapy by reporting on ER function, providing an early indication of response (Fowler et al., 2012). PR imaging has been evaluated using the steroidal PR ligand, 21-[^18^F]fluorofuranyl-norprogesterone ([^18^F]FFNP) in primary breast carcinoma as well as reporting on treatment response in subsequent in vivo animal studies (Chan et al., 2015, Dehdashti et al., 2012, Fowler et al., 2012). Although promising, 20-keto steroidal compounds exhibit cross-reactivity with other SHRs (primarily the glucorticoid receptor, GR) and are prone to metabolism by 20α-hydroxysteroid dehydrogenase (20-HSD).(Zhou et al., 2010) The development of non-steroidal radiolabelled ligands based on the established structure-activity relationship (SAR) of the benzoxazin(thi)one pharmacophore of the PR agonist Tanaproget (Fig. 1) that exhibit high affinity and selectivity to PR while avoiding 20-HSD metabolism has also been explored (Fensome et al., 2005, Winneker et al., 2005, Zhang et al., 2005, Zhang et al., 2002, Zhou et al., 2010).

Structural modification to the 1 *N*-position (substitutive nomenclature described in Fig. 1) of the benzoxazin(thi)one core interrupts an important hydrogen bond (Asn719) formed in the LBD, whereas large substituents are tolerated at the C4-position (Fensome et al., 2005, Zhou et al., 2010). Comparing the biological profile of oxo- and thiocarbamates showed a “flip” effect between agonist and antagonist compounds.^18^ The modification of aryl moieties in the C6-position is tolerated and may improve binding to the PR by establishing further hydrogen bonds, e.g. *N*-methyl-2-cyanopyrrole of Tanaproget (Fensome et al., 2005).

The first example of a radiolabelled non-steroidal PR ligand was [^18^F]fluoropropyl-Tanaproget ([^18^F]FPTP, Fig. 1), which showed favourable biodistribution in the uterus of immature estrogen-primed mice (Lee et al., 2010). Derivatising the gem-dimethyl at the C4-position introduced a chiral centre into the molecule however, and the ligand was evaluated as a racemic mixture. In silico evidence suggested that *R-* and *S-* enantiomers may display different binding affinities and therefore further study would be required to evaluate the enantiomerically pure species. [^18^F]Fluoromethyl-Tanaproget ([^18^F]FMTP) bears a fluoromethyl-substituent at the 1 *N*-pyrrole position of Tanaproget and was shown to maintain a high binding affinity to PR in accordance with structure-activity relationship (SAR) data (Merchant et al., 2016). Larger substituents at this position resulted in an inverse relationship between alkyl chain length and relative binding affinity (RBA), limiting substitution to moieties no larger than a single methyl group (Merchant et al., 2016, Zhou et al., 2010). [^18^F]FMTP was radiolabelled using the [^18^F]fluoromethyltosylate prosthetic group and showed promising in vitro cell uptake but poor in vivo metabolic stability. The development of novel radiochemistry methodology to access carbon-11 thiocarbamates allowed the synthesis of [^11^C]Tanaproget from an acyclic precursor; however, in vitro cell studies were unsuccessful due to uptake modulation by multi-drug resistance proteins (MDR) (Haywood et al., 2015, Merchant et al., 2016). The poor performance of [^11^C]Tanaproget and the limited exploration of fluorine-18 radiolabelled non-steroidal PR ligands in the literature prompted further investigation of suitable ligands for imaging PR with PET (Merchant et al., 2016). Here we report the synthesis of a focused library of achiral, non-steroidal PR ligands which were evaluated in vitro for receptor binding. A lead candidate was selected and radiolabelled with fluorine-18 and in vitro receptor binding, in vivo biodistribution, imaging and metabolic stability was evaluated.

## Methods

### General

Reagents and solvents were purchased from Sigma-Aldrich (Gillingham, UK) and used without further purification. Fluorobromomethane (2 M in acetonitrile) was purchased from ABCR (Karlsruhe, Germany). Nitrogen was used as an inert atmosphere for dry reactions. ^1^H, ^13^C and ^19^F NMR spectra were obtained using a Bruker 500 MHz spectrometer operating at room temperature. Chemical shifts (δ) are reported in parts per million (ppm) and residual solvent peaks have been used as an internal reference. Peak multiplicities have been abbreviated as follows: s (singlet), d (doublet), dd (doublet of doublet), ddd (doublet of doublets of doublets), m (multiplet). Accurate mass spectroscopy was carried out by Karl Heaton at The Department of Chemistry Mass Spectrometry Service, University of York using a Bruker microTOF connected to an Agilent 1200 series LC system. Compound purity, radiochemical preparative RP-HPLC and radio-RP-HPLC was carried out using an Agilent Infinity 1260 quaternary pump system equipped with a 1260 diode array (Agilent Technologies, UK). HPLC methods appear in the supporting information. [^18^F]Fluoride was produced via *the* ^18^O(p,n)^18^F reaction using either a GE PETrace cyclotron by 16 MeV irradiation, supplied by Alliance Medical Radiopharmacy LTD (Warwick, UK) or using an ABT BG-75 mini cyclotron (ABT Molecular Imaging, TN, USA) by 6.9 MeV irradiation, supplied by The University of Hull (Hull, UK). Pooled mouse liver microsomes (MLM) (20 mg/mL, female) for radioactive MLM assays where purchased from Corning (Wiesbaden, Germany). Pooled ICR/CD-1 mouse liver microsomes (MLM) (22 mg/ml, female, 400 donors) and human liver microsomes (HLM) (24 mg/ml, mixed gender, 150 donors) for metabolite identification assays were purchased from BioreclamationIVT (Frankfurt Am Main, Germany).

### Chemical synthesis

**Scheme 1:**
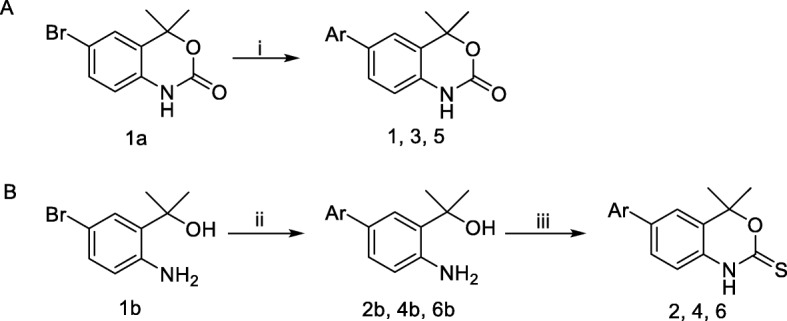
**a** Synthesis towards PR ligands **1**, **3**, **5**. Reaction conditions: (*i*) Pd(PPh_3_)_4_, K_2_CO_3_, EtOH, toluene, aryl boronic acid, 85 °C, 16 h. **b** Synthesis of ligands **2**, **4**, **6**. Reaction conditions: (*ii*) Pd(PPh_3_)_2_Cl_2_, Na_2_CO_3_, MeCN:H_2_O (1:1), μW 150 °C, 6 min; (*iii*) TCDI, THF, 50 °C, 16 h

The procedures for the synthesis of 6-bromo-4,4-dimethyl-1*H*-benzo[*d*][1,3]oxazin-2(4*H*)-one (**1a**), 2-(2-amino-5-bromophenyl)propan-2-ol (**1b**), (4,4-dimethyl-2-oxo-2,4-dihydro-1H-benzo[d][1,3]oxazin-6-yl)boronic acid (**1c**) and Tanaproget were followed from the literature(Zhang et al., 2005). The Suzuki-coupling procedure reported in Scheme 1a was adapted from the literature (Fensome et al., 2005).

*6-(2-fluoropyridin-3-yl)-4,4-dimethyl-1H-benzo[d][1,3]oxazin-2(4H)-one (* ***1*** *).*

Compound **1a** (414 mg, 1.6 mmol) and Pd(PPh_3_)_4_ (47 mg, 0.04 mmol) were stirred in toluene (8 mL) under an inert atmosphere. 6-Fluoropyridine-5-boronic acid (624 mg, 3.8 mmol) was dissolved in EtOH (8 mL) and added to the stirred flask, followed by K_2_CO_3_ (436 mg, 3.1 mmol) dissolved in H_2_O (8 mL). The reaction mixture was degassed and heated to 85 °C under an inert atmosphere for 16 h. The reaction was cooled, quenched with sat. NaHCO_3_ (10 mL) and extracted with EtOAc (3 × 40 mL). Organic fractions were combined and washed with brine (100 mL), water (100 mL) and dried over anhyd. MgSO_4_. Solvent was removed in vacuo, the product was isolated by column chromatography (silica gel, 80% EtOAc / 20% hexane) as a white solid (22 mg, 5%). ^1^H NMR (500 MHz, CDCl_3_) δ 9.44 (s, 1H), 8.21 (ddd, *J* = 4.9, 1.9, 1.1 Hz, 1H), 7.86 (ddd, *J* = 9.7, 7.4, 2.0 Hz, 1H), 7.46 (dt, *J* = 8.2, 1.6 Hz, 1H), 7.37 (t, *J* = 1.7 Hz, 1H), 7.30 (ddd, *J* = 7.4, 4.8, 1.7 Hz, 1H), 7.02 (d, *J* = 8.2 Hz, 1H), 1.80 (s, 6H). ^13^C NMR (126 MHz, CDCl_3_) δ 161.21, 159.30, 152.92, 146.32, 140.29, 134.17, 130.53, 126.75, 123.83, 123.07, 121.91, 115.04, 83.05, 28.13. ^19^F NMR (471 MHz, CDCl_3_) δ − 71.10. HRMS: calc’d for C_15_H_14_FN_2_O_2_, 273.1034; found (ESI), 273.1025 [(M + H)^+^].

*2-(2-amino-5-(2-fluoropyridin-3-yl)phenyl)propan-2-ol (* ***2b*** *).*

Compound **1b** (200 mg, 0.9 mmol), (6-fluoropyridin-3-yl)boronic acid (147 mg, 1.0 mmol), Na_2_CO_3_ (184 mg, 1.7 mmol) and Pd(PPh_3_)_2_Cl_2_ were added to a microwave tube and dissolved in degassed MeCN:H_2_O (1:1 *v*/v, 9 mL). The vessel was heated in a microwave to 150 °C (200 W) for 6 min. The reaction mixture was poured into water (30 mL) and extracted with EtOAc (3 × 50 mL). Organic fractions were combined and dried over anhyd. MgSO_4_. Solvent was removed in vacuo and purified by column chromatography (silica, 40% EtOAc / 60% hexane) to give product as a brown solid (131 mg, 61%). ^1^H NMR (CDCl_3_, 400 MHz) δ 8.10–8.11 (m, 1H), 7.80–7.83 (m, 1H), 7.36 (m, 1H), 7.31 (m, 1H), 7.21–7.24 (m, 1H), 6.72 (d, 1H, *J* = 8.4 Hz), 1.72 (s, 6H). ^13^C NMR (CDCl_3_, 100 MHz) δ 29.2, 74.1, 117.3, 121.7, 123.3, 126.3, 128.5, 130.4, 139.78, 144.6, 146.2, 159.1, 161.5. ^19^F NMR (CDCl_3_ 400 MHz) δ 71.25. HRMS: calc’d for C_14_H_16_FN_2_O, 247.1241; found (ESI), 247.1239 [(M + H)^+^].

*6-(2-fluoropyridin-3-yl)-4,4-dimethyl-1H-benzo[d][1,3]oxazine-2(4H)-thione (* ***2*** *).*

Compound **2a** (250 mg, 1.0 mmol) and 1,1′-thiocarbonyldiimidazole (217 mg, 1.2 mmol) were stirred in dry THF (50 mL) at 50 °C under an inert atmosphere for 16 h. Bulk solvent was removed in vacuo and the crude material was dissolved in EtOAc (50 mL) and washed with aqueous HCl (1 M). Organic fractions were combined and dried over anhyd. MgSO_4_. Solvent was removed in vacuo and the product was isolated by column chromatography (silica, 40% EtOAc / 60% hexane) followed by precipitation from ether to give product (65 mg, 22%). ^1^H NMR (500 MHz, CDCl_3_) δ 9.67 (s, 1H), 8.24 (ddd, *J* = 4.9, 1.9, 1.2 Hz, 1H), 7.86 (ddd, *J* = 9.7, 7.4, 2.0 Hz, 1H), 7.51 (dt, *J* = 8.3, 1.6 Hz, 1H), 7.39 (t, *J* = 1.6 Hz, 1H), 7.32 (ddd, *J* = 7.4, 4.9, 1.7 Hz, 1H), 7.01 (d, *J* = 8.2 Hz, 1H), 1.83 (s, 6H). ^13^C NMR (126 MHz, CDCl_3_) δ 184.17, 146.78, 146.66, 140.29, 131.37, 131.03, 129.59, 127.16, 123.89, 122.68, 121.98, 114.3, 84.81, 27.73. ^19^F NMR (471 MHz, CDCl_3_) δ − 70.96. HRMS: calc’d for C_15_H_14_FN_2_OS, 289.0805; found (ESI), 289.0800 [(M + H)^+^].

*6-(6-fluoropyridin-3-yl)-4,4-dimethyl-1H-benzo[d][1,3]oxazin-2(4H)-one (* ***3*** *).*

Compound **3** was synthesised in a similar way to compound **1** in a 29% yield. ^1^H NMR (500 MHz, CDCl_3_) δ 9.03 (s, 1H), 8.39 (dt, *J* = 2.5, 0.8 Hz, 1H), 7.94 (ddd, *J* = 8.4, 7.5, 2.6 Hz, 1H), 7.43 (dd, *J* = 8.2, 2.0 Hz, 1H), 7.03 (ddd, *J* = 8.4, 3.1, 0.7 Hz, 1H), 7.00 (d, *J* = 8.2 Hz, 1H), 1.81 (s, 6H). ^13^C NMR (126 MHz, CDCl_3_) δ 164.03, 162.13, 152.57, 145.55, 139.46, 135.55, 132.33, 127.74, 127.31, 122.00, 115.33, 109.56, 82.93, 28.10. ^19^F NMR (471 MHz, CDCl_3_) δ − 70.26. HRMS: calc’d for C_15_H_14_FN_2_O_2_, 273.1034; found (ESI), 273.1025 [(M + H)^+^].

*2-(2-amino-5-(6-fluoropyridin-3-yl)phenyl)propan-2-ol (* ***4b*** *).*

Compound **4b** was synthesised in a similar way to compound **2b** in a 61% yield. ^1^H NMR (CDCl_3_, 400 MHz) δ 8.31 (d, 1H, *J* = 2.5 Hz), 7.85–7.90 (m, 1H), 7.28 (d, 1H, *J* = 2.2 Hz), 7.23 (d, 1H, *J* = 2.2 Hz), 6.95 (dd, 1H, *J* = 3.1, 8.6 Hz), 6.74 (d, 1H, *J* = 8.2 Hz), 1.73 (s, 6H). ^13^C NMR (CDCl_3_, 100 MHz) δ 29.1, 73.7, 109.0, 117.5, 125.1, 125.2, 127.9, 131.2, 135.1, 143.0, 145.9, 160.9, 163.3. ^19^F NMR (CDCl_3_ 400 MHz) δ 72.64. HRMS: calc’d for C_14_H_16_FN_2_O, 247.1241; found (ESI), 247.1235 [(M + H)^+^].

*6-(6-fluoropyridin-3-yl)-4,4-dimethyl-1H-benzo[d][1,3]oxazine-2(4H)-thione (* ***4*** *).*

Compound **4** was synthesised in a similar way to compound **2** in a 19%. ^1^H NMR (500 MHz, CDCl_3_) δ 9.47 (s, 1H), 8.40 (dt, *J* = 2.7, 0.8 Hz, 1H), 7.94 (ddd, *J* = 8.5, 7.5, 2.6 Hz, 1H), 7.48 (dd, *J* = 8.2, 2.0 Hz, 1H), 7.31 (d, *J* = 2.0 Hz, 1H), 7.04 (ddd, *J* = 8.5, 3.1, 0.7 Hz, 1H), 6.99 (d, *J* = 8.2 Hz, 1H), 1.83 (s, 6H). ^13^C NMR (126 MHz, CDCl_3_) δ 184.10, 145.66, 139.49, 134.06, 133.70, 131.31, 127.91, 127.67, 122.04, 114.66, 109.85, 109.55, 84.69, 27.69. ^19^F NMR (471 MHz, CDCl_3_) δ − 69.63. HRMS: calc’d for C_15_H_14_FN_2_OS, 289.0805; found (ESI), 289.0815 [(M + H)^+^].

*6-(3-chloro-4-fluorophenyl)-4,4-dimethyl-1H-benzo[d][1,3]oxazin-2(4H)-one (* ***5*** *).*

Compound **5** was synthesised in a similar way to compound **1** in a 41% yield. ^1^H NMR (500 MHz, MeOD-*d*_4_) δ 7.72 (dd, *J* = 7.0, 2.4 Hz, 1H), 7.56 (ddd, *J* = 8.6, 4.5, 2.3 Hz, 1H), 7.52 (dd, *J* = 8.2, 2.0 Hz, 1H), 7.49 (d, *J* = 2.0 Hz, 1H), 7.31 (t, *J* = 8.9 Hz, 1H), 6.99 (d, *J* = 8.2 Hz, 1H), 1.75 (s, 6H). ^13^C NMR (126 MHz, MeOD-*d*_4_) δ 167.95, 156.40, 152.63, 137.92, 134.15, 134.07, 128.40, 127.14, 126.57, 121.78, 120.77, 116.53, 114.61, 82.47, 26.79. ^19^F NMR (471 MHz, MeOD-*d*_4_) δ − 120.80. HRMS: calc’d for C_16_H_14_ClFNO_2_, 306.0692; found (ESI), 306.0692 [(M + H)+].

*2-(4-amino-3′-chloro-4′-fluoro-[1,1′-biphenyl]-3-yl)propan-2-ol (* ***6b*** *).*

Compound **6b** was synthesised in a similar way to compound **2b** in a 67% yield. ^1^H NMR (400 MHz, CDCl_3_) δ 7.49 (ddd, *J* = 7.0, 2.3, 0.8 Hz, 1H), 7.35–7.29 (m, 1H), 7.27–7.24 (m, 1H), 7.21 (ddd, *J* = 8.1, 2.2, 0.8 Hz, 1H), 7.13 (td, *J* = 8.7, 0.7 Hz, 1H), 6.68 (dd, *J* = 8.2, 0.7 Hz, 1H), 1.70 (s, 6H). ^13^C NMR (400 MHz, CDCl_3_) δ 171.37, 158.22, 155.76, 145.70, 138.88, 130.99, 128.35, 126.85, 126.03, 124.50, 117.86, 116.72, 74.30, 29.44. ^19^F NMR (500 MHz, CDCl_3_) δ − 119.80. HRMS: calc’d for C_15_H_16_ClFNO, 280.0899; found (ESI), 280.0898 [(M + H)^+^].

*6-(3-chloro-4-fluorophenyl)-4,4-dimethyl-1H-benzo[d][1,3]oxazine-2(4H)-thione (* ***6*** *).*

Compound **6** was synthesised in a similar way to compound **2** in a yield of 30%. ^1^H NMR (500 MHz, CDCl_3_) δ 9.62 (s, 1H), 7.56 (dd, *J* = 6.9, 2.3 Hz, 1H), 7.45 (dd, *J* = 8.2, 2.0 Hz, 1H), 7.39 (ddd, *J* = 8.5, 4.5, 2.3 Hz, 1H), 7.29 (d, *J* = 1.9 Hz, 1H), 7.23 (t, *J* = 8.6 Hz, 1H), 6.97 (d, *J* = 8.2 Hz, 1H), 1.83 (s, 6H). ^13^C NMR (126 MHz, CDCl_3_) δ 184.05, 158.82, 156.83, 137.20, 136.45, 130.96, 129.01, 127.78, 127.41, 126.54, 123.26, 117.05, 114.53, 84.79, 27.74. ^19^F NMR (471 MHz, CDCl_3_) δ − 117.17. HRMS: calc’d for C_16_H_14_ClFNOS, 322.0463; found (ESI), 322.0459 [(M + H)^+^].

*4,4-dimethyl-6-(2-nitropyridin-3-yl)-1H-benzo[d][1,3]oxazin-2(4H)-one (* ***7*** *).*

Compound **1c** (200 mg, 0.90 mmol), 2-nitro-3-bromopyridine (164 mg, 0.80 mmol), K_2_CO_3_ (226 mg, 1.63 mmol) and Pd(PPh_3_)_2_Cl_2_ (28 mg, 0.04 mmol) were added to a microwave tube (10 mL) and dissolved in degassed MeCN:H_2_O (1:1, 7 mL). The vessel was heated in the microwave (150 °C, 200 W) for 20 min. The reaction mixture was allowed to cool, poured into water (30 mL) and extracted with EtOAc (3 × 50 mL). Organic fractions were combined and dried over anhyd. MgSO_4_. Solvent was removed in vacuo and product was isolated by column chromatography (silica, 80% EtOAc / 20% hexane) as a beige solid (130 mg, 48%). ^1^H NMR (500 MHz, CDCl_3_) δ 8.64 (s, 1H), 8.54 (dd, *J* = 4.7, 1.7 Hz, 1H), 7.93 (dd, *J* = 7.7, 1.7 Hz, 1H), 7.66 (dd, *J* = 7.7, 4.7 Hz, 1H), 7.26 (dd, *J* = 2.0 Hz, 1H), 7.14 (d, *J* = 2.0 Hz, 1H), 6.96 (d, *J* = 8.2 Hz, 1H), 1.76 (s, 6H). ^13^C NMR (126 MHz, CDCl_3_) δ 157.63, 152.08, 150.04, 147.46, 141.06, 134.84, 129.49, 129.15, 128.74, 127.21, 127.16, 123.10, 115.14, 82.83, 28.01.

### Radiosynthesis of **[**^**18**^**F]2**

To a Wheaton vial was added a mixture of K_2_CO_3_ (0.1 M, 100 μL), Kryptofix™ K_222_ (3 mg), acetonitrile (200 μL) and [^18^F]fluoride (1.5–1.7 GBq in 200–300 μL ^18^O-water). The solvent was removed by azeotropic distillation at 110 °C under a stream of nitrogen with three repeat additions of acetonitrile (300 μL) until dry. A solution of precursor **7** (1 mg, dissolved in 300 μL dry DMSO) was added to the vial and heated at 160 °C for 15 min. An aliquot (2 μL) was taken for RP-HPLC analysis (Additional file 1: Figure S28) using gradient 2 (supporting information, section 5). The reaction mixture was added to water (10 mL) and immobilized on a Sep Pak C18 light cartridge (pre-conditioned with MeOH (5 mL) and water (10 mL)). The cartridge was washed with water (5 mL) to remove residual [^18^F]fluoride and **[**^**18**^**F]1** was eluted with MeOH (700–1000 μL) into a clean Wheaton vial. The MeOH was removed by evaporation under a stream of nitrogen (*ca* 5 min) until a residue remained. Lawessons reagent (15 mg) was added to dry residue followed by toluene (300 μL). The vial was sealed tightly and heated to 135 °C for 35 min. The toluene was evaporated under a stream of nitrogen (*ca* 5 min) and the reaction mixture was reconstituted into DMSO (400 μL). An aliquot (5 μL) was taken for RP-HPLC analysis (Additional file 1: Figure S29) using gradient 3 (supporting information, section 5). The reaction mixture was filtered and purified using preparative RP-HPLC. The cut peak was diluted in water (10 mL) and immobilized on a HLB cartridge (10 cc), pre-conditioned with EtOH (5 mL) and water (10 mL). The immobilized product was washed with water (5 mL) and eluted with EtOH (400 μL) into a clean vial. The EtOH was evaporated to ~ 30 μL volume and diluted with PBS to give a final solvent composition of 10% EtOH/PBS for biological use. An aliquot (20 μL) was taken for RP-HPLC analysis (Additional file 1: Figures S9 - S10).

### Distribution coefficient analysis (LogD_7.4_)

Radioligand **[**^**18**^**F]2** (0.03 MBq, 1 μL, in EtOH) was mixed with PBS (500 μL) and *n*-octanol (500 μL). The sample was vortexed (10 min) followed by centrifugation (1×g, 10 min). Three aliquots (100 μL) from each layer were taken and counts measured using a 2480 WIZARD2 Automatic Gamma Counter (PerkinElmer, UK). Experiment was repeated in triplicate in *n* = 3 determinations (supporting information, Additional file 1: Table S1). Distribution coefficient (LogD_7.4_) was calculated as the logarithm of the ratio between CPM of the octanol and buffer phase.

### T47D potency studies

The method described by Di Lorenzo et al was followed with minor adaptation (Di Lorenzo et al., 1991). In brief, T47D breast carcinoma cells were seeded in 96-well plates at 50,000 cell/well in RPMI medium supplemented with 10% FBS. After overnight culture, the medium was changed to RPMI phenol red free containing 2% charcoal-stripped FBS. After 24 h, the cells were treated with progesterone (Sigma Aldrich, Gillingham, UK) or the library compounds. Progesterone and the test compounds were dissolved in DMSO (100%) and diluted into treatment medium to give a final DMSO (*v*/v) concentration of 0.1%. After incubation (48 h) the treatment was finalised by twice washing the plates with PBS. Cells were lysed by two freeze-thaw cycles (− 80 °C). Cellular alkaline phosphatase activity was determined by adding Femto ELISA-AP substrate (200 μL) and optical density measurements taken at 5 and 10 min intervals at a wavelength of 405 nm. Data was interpreted using Graphpad Prism (GraphPad, CA, USA) using the model: dose-response simulation/inhibition, log(agonist/inhibition) vs. response – variable slope (four parameters). For compounds where EC_50_ > 10,000, an antagonist profile was assumed and the experiment was repeated with the inclusion of progesterone (3 mM) with the library compound. Data shown in the supporting information (Additional file 1: Figure S32).

### GR nuclear translocation assay

Compound **2** was analysed for GR binding using the PatHunter® express CHO-K1 GR Nuclear Translocation assay (DiscoveRx™, Fremont, CA, USA). Reagents were thawed and equilibrated to room temperature prior to use. Dexamethasone standard (10 mM, 100% DMSO) was diluted and used to prepare 12 standards by 1:4 dilution. Test compound **2** was dissolved in 100% DMSO and diluted in serial 1:3 dilutions to a final concentration of DMSO (v/v) of 0.1%. CHO-K1 (Chinese hamster ovary) cells were seeded in a 96-well plate with CP reagent and incubated for 24 h at 37 °C, 5% CO_2_. Cells were then incubated with the Dexamethasone standard and compound **2** for 6 h at 37 °C, 5% CO_2_. Following incubation, signal was detected using the PathHunter® detection reagent provided in the kit and incubated in the dark for 1 h. The plate was read using a luminescence plate reader (Additional file 1: Figure S33).

### Immunoblotting

Protein samples were analysed by sodium dodecyl sulfate-polyacrylamide (SDS-PAGE) gel followed by Western blotting. Equal amount of proteins per sample (50–100 μg) was resolved on a 10%–12% SDS-PAGE. Molecular weight ladders (Precision Plus Protein Western C Standard, BioRad, CA, USA) were loaded together with the samples. Electrophoresis was performed at 100-120 V for 1.5 h. When the migration front reached the bottom of the gel, electrophoresis was stopped and proteins were transferred onto a 0.45-μm PVDF membrane (polyvinylidene difluoride) previously activated in methanol for 2 min. The transfer was performed for 1 h at 100 V in 1× blotting buffer containing 20% methanol. The PVDF membrane was blocked for 1 h in a milk solution (5% fat-free powdered milk in 0.1% PBST and 0.1% Tween) and rinsed thoroughly in 0.1% PBST. The membrane was incubated overnight at 4 °C with the primary antibody (PR antibody from Cell Signalling) in a 1:1000 dilution in 5% milk-0.1% PBST. Membrane was washed 3 times (5 min/wash) in 0.1% PBST and incubated 1 h at room temperature with the appropriate secondary antibody (anti-rabbit IgG 1:2000 dilution). The membrane was washed 3 more times in 0.1% PBST, and incubated with 1 mL of reagent mixture (1:1) form the Amersham enhanced chemiluminescence (ECL) Plus Western Blotting Detection kit (GE Healthcare) and imaged using the ChemiDoc XRS+ System and the Image Lab software (BioRad, CA, USA).

### Cell uptake study

T47D, MCF-7 and MDA-MB-231 cells (1 × 10^5^) were plated into 6-well plates and used for receptor binding assays at 60–80% confluence. Cell medium was removed and cells were washed with cold PBS buffer. **[**^**18**^**F]2** (37 kBq/mL) was added to individual wells and cells were incubated at 37 °C in a humidified atmosphere of 5% CO_2_ for 60 min. Cells were co-incubated with 0.01–1 μM of cold progesterone for the blocking study. Cells were then washed 3 times with ice-cold PBS and lysed with RIPA buffer. Radioactivity was determined using a gamma spectrometer (Automatic Gamma counter, Wizard 3 Wallac). Radioactivity was normalised to protein content determined using a BCA 96-well plate assay (Thermo Fisher Scientific Inc., USA) and expressed as a percentage of total radioactivity per mg of protein.

### Mouse model

Pubertal female C57BL/6 mice were injected intraperitoneally with 30 IU gonadotropins, 15 IU Follicle-stimulating hormone (FSH) and 15 IU luteinizing hormone (LH) followed by 5 IU menotropin (hCG) after 48 h (Sigma-Aldrich, Gillingham, UK). After the imaging studies, the ovaries were removed and snap-frozen in liquid nitrogen for later analysis of protein expression by western blot.

### Biodistribution

**[**^**18**^**F]2** (455 ± 62 kBq) was injected into the tail vail of female C57BL/6 mice. Whilst under anaesthesia the animals were sacrificed and tissues of interest such as, heart, lung, liver, spleen, kidneys, stomach, muscle, bone, ovary, small intestines and large intestines were collected in pre-weighed counting tubes. Radioactivity within tissue samples was counted in a gamma spectrometer (Automatic Gamma Counter, Wizard 3 Wallac), and then weighed to determine the mass of the tissue. CPM for each tissue sample was normalised to the total injected dose of radioactivity to the animal to give %ID (injected dose), and then normalised to the weight of the counted tissue to give the radioactivity uptake of the tissue as %ID/g. Tabulated values shown in Additional file 1: Table S2.

### PET/CT imaging

Mice were anaesthetised with 3–5% isoflurane at a flow rate of 1 L/min, and then reduced to 1.5–2% isoflurane for anaesthesia maintenance during imaging. **[**^**18**^**F]2** was injected via the tail vein at the commencement of a dynamic PET scan using a dedicated small animal PET/CT scanner (Sedecal SuperArgus2R, Sedecal, Spain). Images were acquired over a 50 min imaging sequence, followed by CT imaging acquired in 514 projections for anatomic coregistration. PET emission data were corrected for decay and dead time and reconstructed using 3D-OSEM. Data were analysed based on regions of interest (ROI) drawn within the tumours and tissue. Time-activity curves (TAC) were calculated as an average form the region of interest analysis of three mice (Additional file 1: Figure S34). Data was analysed using AMIDE software (Loening and Gambhir, 2003) and VivoQuant (InVicro, MA, USA) and regions of interest (ROIs) were selected by hand, and the count densities were averaged for each frame to obtain a TAC for each ROI.

### In vitro radioactive metabolite analysis of **[**^**18**^**F]2**

Radioligand **[**^**18**^**F]2** (3 MBq, ca. 80 μL), mouse liver microsomes (50 μL, 1 mg/mL), NADPH regeneration system A (50 μL), NADPH regeneration system B (10 μL), PBS 0.5 M pH 7.5 (200 μL) and water (ca. 200 μL) were added to a plastic 1.5 mL tube. The mixture was incubated at 37 °C for 60 min and transferred into a plastic centrifuge tube (10 mL). Proteins were precipitated with ice-cold MeOH (2 mL) and the mixture was centrifuged (12,000 g, 3 min) to pellet the precipitate. The supernatant was removed, evaporated to dryness and the residue was suspended in 37% MeCN + 0.1%TFA / 63% Water + 0.1%TFA for RP-HPLC using gradient 3 (supporting information, section 10.3). The experiment was performed in *n* = 3 determinations. Extraction efficiency of radioactivity from the protein pellet was calculated to be 93.5% ± 1.6 (Additional file 1: Table S3). HPLC data is shown in the supporting information (Additional file 1: Figure S35).

### In vitro non-radioactive metabolite analysis of **2**

Microsomal incubations were performed using a Hamilton Microlab Star liquid handling workstation (Hamilton Robotics, Bonaduz, Switzerland). Test compound **2** (final concentration 1 or 10 μM, 1% DMSO) was pre-incubated at 37 °C for 10 min in MLM or HLM (final concentration 1 mg/ml prepared in 10 mM PBS). Reactions were initiated by the addition of NADPH (final concentration 1 mM). Aliquots were removed from each incubation and quenched in 3 volumes of ice-cold MeOH containing internal standard olomoucine (500 nM in MeOH) at − 1, 0, 5, 15, 30 and 60 min. Incubations were conducted in singlicate. Inactive control incubations (without NADPH) were conducted in parallel. Terminated incubations were centrifuged at 3700 rpm at 4 °C for 30 min and supernatant taken for analysis. Microsomal incubations were analysed by liquid chromatography-tandem mass spectrometry (LC-MS/MS). This consisted of a Dionex Ultimate 3000 UHPLC system coupled to a ThermoScientific Q Exactive Plus orbitrap mass spectrometer (Thermo Fisher Scientific Inc., Waltham, USA). Chromatographic separation was achieved using an Acquity UPLC HSS T3 column (1.8 μm, 100 × 2.1 mm) (Waters, Elstree, UK) at 50 °C and a binary mobile phase gradient at a flow rate of 0.4 ml/min. Initial LC conditions comprised 10% formic acid (0.1%) in water (A), 90% methanol (B). This was ramped to 95% A at 12 min. Sample analysis was by positive ion electrospray ionization. The capillary voltage was 3.5 kV. Full MS/dd-MS^2^ (full MS scan followed by data dependent MS/MS) and Full MS/AIF (full MS scan followed by all ion fragmentation) workflows were used in combination. Full MS was performed at a resolution of 70,000, AGC target 1 e^6^; dd-MS2 at a resolution of 17,5000, AGC target 1 e^5^. LC-MS response (peak area of the analyte/peak area of internal standard) was fitted to a nonlinear single exponential model using software GraphPad Prism software (v6.07, GraphPad Software, Inc., La Jolla, USA). Elimination rate constant (k) was determined and intrinsic clearance, CL_int_, was calculated as CL_int_ = (V.k)/mg protein where V is the incubation volume (μl) and mg protein is the mg microsomal protein in the incubation. Identification of metabolites in microsomal incubations and elucidation of their structures was undertaken with Compound Discoverer software (v2.0.0.303, Thermo Fisher Scientific Inc., Waltham, USA).

## Results

### Chemistry

**Fig. 2:**
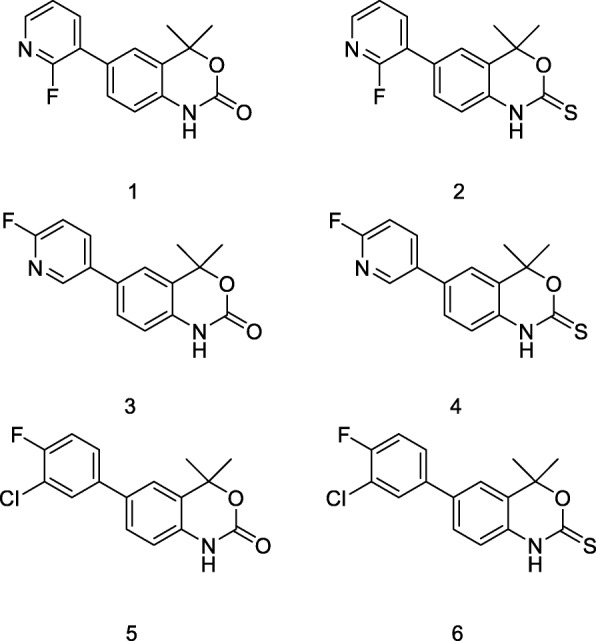
Focused library of PR ligands (**1**–**6**)

A focused library of benzoxazin(thi)one compounds (Fig. 2) was synthesised using two routes: Route A (Scheme 1a), where benzoxazinone compounds were synthesised from aryl-bromide **1a** using palladium catalysed Suzuki-coupling chemistry with commercially available fluorine-substituted boronic acids; compounds **1**, **3** and **5** were accessed in *ca* 18% yield. Benzoxazinthione compounds were synthesised using Route B (Scheme 1b), an “acyclic approach” employing a Suzuki-coupling reaction with aryl-bromide **1b** and fluoro-aryl boronic acids to form biaryl “acyclic compounds” (**2b, 4b, 6b**) in *ca* 63% yield. Subsequent installation of the thiocarbamate using 1,1′-thiocarbonyldiimidazole (TCDI) yielded compounds **2**, **4** and **6** in an overall yield of *ca* 18%. Compounds were characterised by ^1^H/^13^C/^19^F-NMR (Additional file 1: Figures S1 S20), HRMS and compound purity was > 95% by RP-HPLC (Additional file 1: Figures S21 S27).

### In vitro potency assay

Compounds (**1**–**6**) were evaluated in an T47D alkaline phosphatase (AP) assay to identify a lead candidate for further evaluation (Table 1). Tanaproget was included as a positive control and the EC_50_ was comparable with the literature (T47D EC_50_ = 0.15 nM) (Fensome et al., 2005).

**Table 1:** Potency of **1**–**6** in a T47D alkaline phosphatase assays, including Tanaproget (TNP) as a reference compound

compd	cLogP^*c*^	T47D alkaline phosphatase assay EC_50_ (nM)^*a*^	T47D alkaline phosphatase assay IC_50_ (nM)^*a*^
**TNP**	4.16	0.5	–
**1**	2.98	> 10,000^*b*^	844.8 ± 0.23
**2**	4.17	4.7 ± 0.07	–
**3**	2.98	> 10,000^*b*^	795.0 ± 0.25
**4**	4.17	3674.0 ± 0.08	–
**5**	5.02	2294.0 ± 0.29	–
**6**	6.18	432.5 ± 0.24	–

^a^Experimental values for **1–6** are presented as an average of at least n = 3 measurements ± standard deviation (SD).^*b*^ The EC_50_ of **1** and **3** showed no agonist activity so IC_50_ values were calculated from the inhibition of progesterone (3 mM). ^*c*^ Calculated log *P*_*o/w*_ from Chemdraw 12.0

### Radiochemistry

**Scheme 2:**
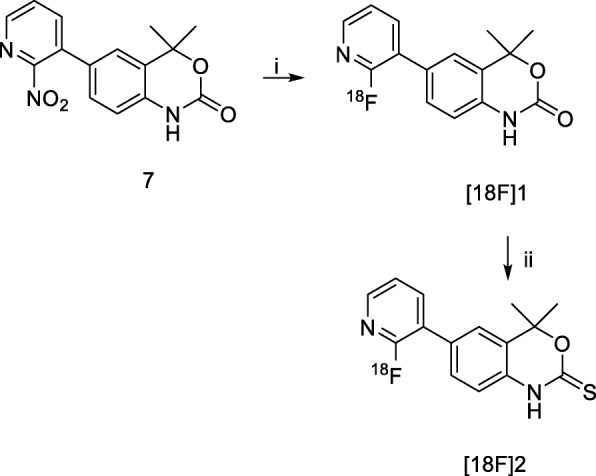
Radiosynthesis of **[**^**18**^**F]2**; Reaction conditions: (*i*) K[^18^F]fluoride/K_2.2.2_, K_2_CO_3_, DMSO, 160 °C 15 min; (*ii*) Lawessons reagent (15 mg), toluene, 135 °C, 35 min

The nitro-containing precursor (**7**) was synthesised by conversion of **1a** into a boronic acid **1c** followed by palladium catalysed Suzuki-coupling with 3-bromo-2-nitropyridine (Additional file 1: Figure S1). The radiolabelling of **[**^**18**^**F]2** was achieved by [^18^F]fluoride incorporation into precursor (**7**) with K[^18^F]fluoride/K_2.2.2_ and K_2_CO_3_ to yield **[**^**18**^**F]1** followed by conversion to the thiocarbamate (**[**^**18**^**F]2**) using Lawessons reagent (Scheme 2); average [^18^F]fluoride incorporation into **[**^**18**^**F]1** was 60.9 ± 14.1% (*n* = 5) after 15 min. Solvent was exchanged from DMSO to toluene, assisted by C-18 solid-phase extraction (SPE), followed by the addition of Lawessons reagent to convert the intermediate **[**^**18**^**F]1** into the desired thiocarbamate **[**^**18**^**F]2**, with an efficiency of 43.9 ± 23.1% (n = 5, Additional file 1: Figure S9). Compound **[**^**18**^**F]2** was isolated by preparative RP-HPLC and reformulated using HLB-SPE into 10% (*v*/v) EtOH/PBS for biological evaluation with a molar activity of 2.5 ± 1.6 GBq/μmol (mean ± SD) and a radiochemical purity ≥95%. The identity of **[**^**18**^**F]2** was confirmed by HPLC co-elution with an authentic sample of compound **2** (Additional file 1: Figure S30). The radiochemical yield (decay-corrected to the start of synthesis) was 2.29 ± 2.31% (*n* = 6) with a synthesis time of 167 min (n = 6). Radioligand **[**^**18**^**F]2** was 99.7% stable up to 4 h after formulation. The distribution coefficient (LogD_7.4_) was determined by partitioning **[**^**18**^**F]2** between PBS (pH 7.4) and octan-1-ol and was calculated to be 1.69 ± 0.1 (Additional file 1: Table S1).

### In vitro cell uptake assay

**Fig. 3:**
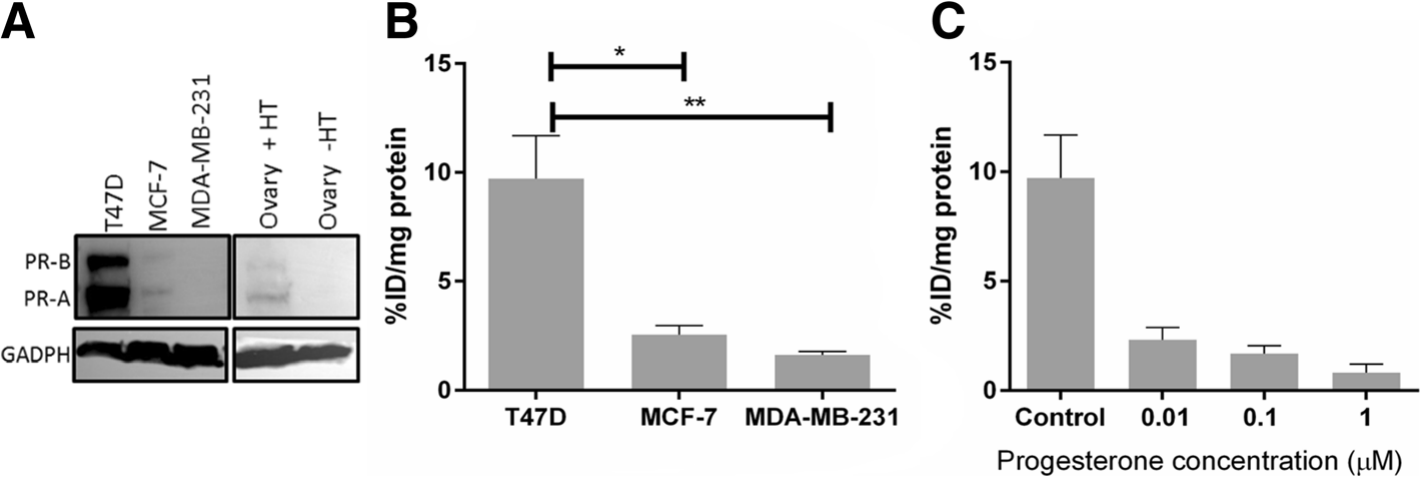
In vitro characterisation of **[**^**18**^**F]2** in breast cancer cell lines with varying PR expression levels. **a** Western Blot showing PR expression in cell lines and in ovarian of C57BL/6 female mice with or without gonadotropic stimulation of PR expression (+/− HT). **b** Uptake of **[**^**18**^**F]2** in three cell lines. **c** Blocking **[**^**18**^**F]2** uptake in T47D cells treated with progesterone (0.01–1 μM). Error bars indicate standard errors from *n* = 3 repeats. Significances are marked with asterisk, (*) and (**) *P* ≤ 0.05, *P* ≤ 0.01, respectively

The uptake of **[**^**18**^**F]2** was evaluated in a panel of three breast cancer cell lines with varying PR expression, determined by Western Blot analysis (T47D = PR++, MCF-7 = PR+ and MDA-MB-231 = PR-, Fig. 3a). Radioligand **[**^**18**^**F]2** showed significant accumulation in T47D cells (PR++) compared to MCF7 (PR+) and MDA-MB-231 (PR-) cells, suggesting a PR-mediated uptake (Fig. 3b). The uptake was confirmed to be specific in T47D cells by co-incubating **[**^**18**^**F]2** with progesterone (0.01–0.1 μM). The uptake of **[**^**18**^**F]2** decreased in a dose-dependent manner (Fig. 3c) which confirmed that **[**^**18**^**F]2** was occupying the LBD of PR.

### In vivo biodistribution

**Fig. 4:**
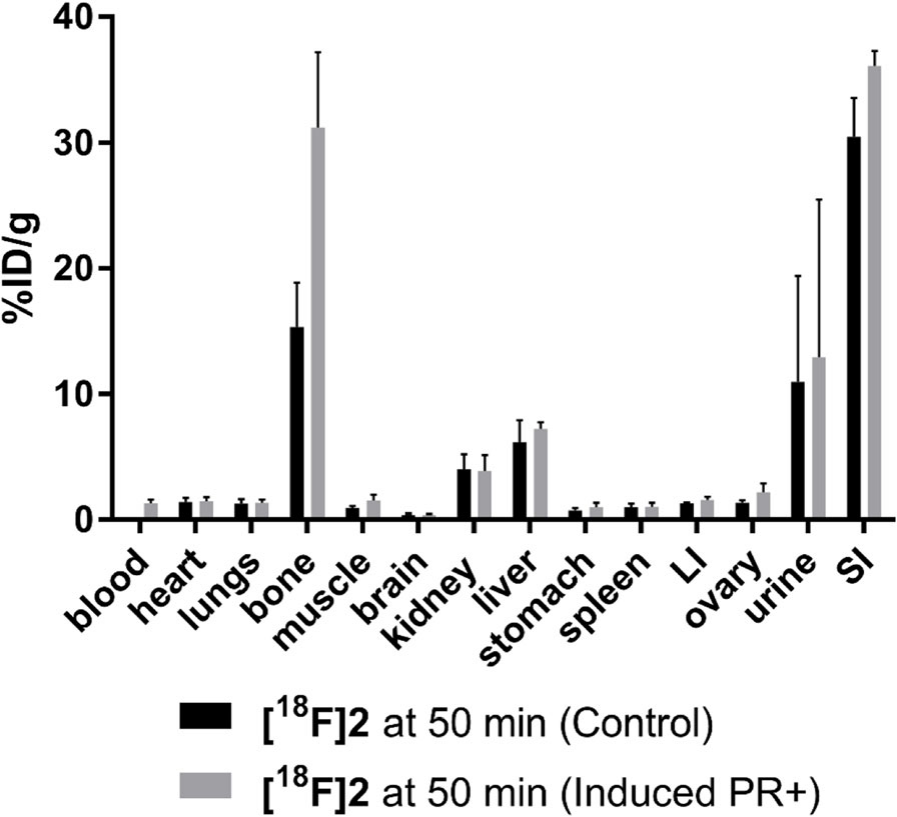
Biodistribution analysis of selected tissues from C57BL/6 female mice injected with **[**^**18**^**F]2** and sacrificed at 50 min post-injection. A group of four animals received a dose of gonadotropins (15 IU FSH + 15 IU LH) plus 5 IU hCG, 48 and 6 h, respectively, before the imaging studies. All radioactivity values were converted in %ID/g of tissue. Biodistribution data are means of ± SEM of three to four animals. LI = large intestine, SI = small intestine. Tabulated values shown in Additional file 1: Table S2

**Fig. 5:**
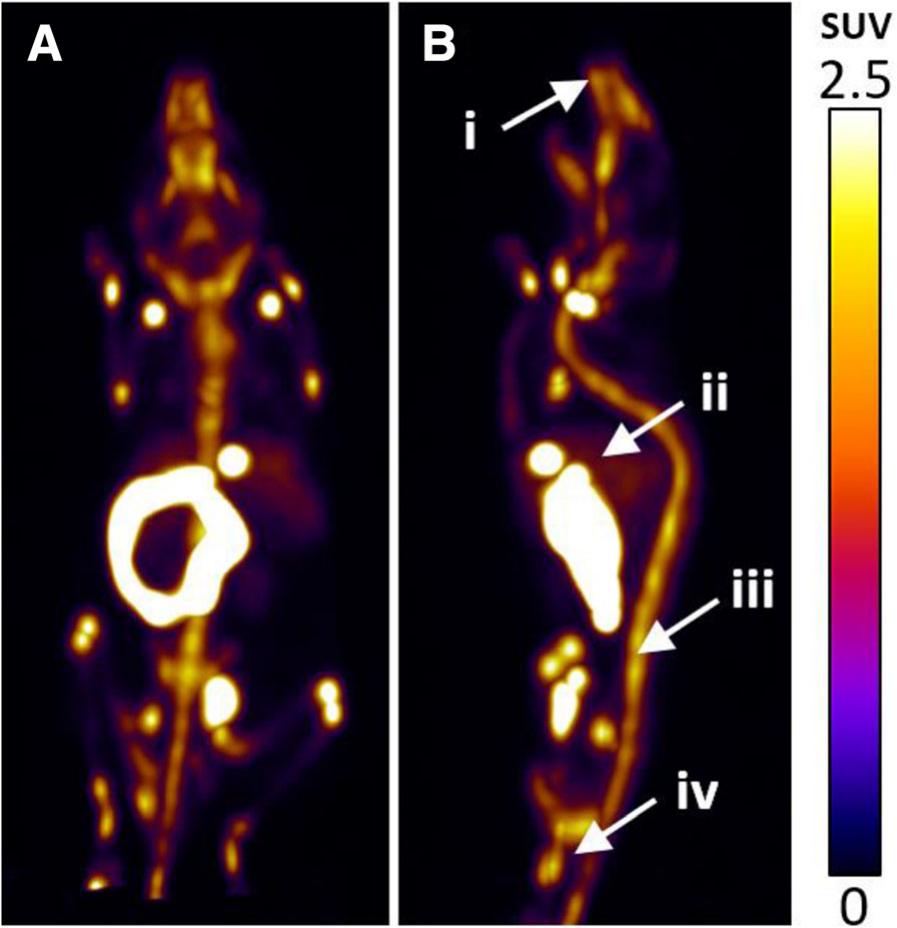
Representative coronal (**a**) and sagittal (**b**) PET/CT images of [^18^F]2 in C57BL/6 female mice at 50 min p.i. White arrows indicate the bone (i), liver (ii), small intestine (iii) and muscle (iv). Extensive skeletal uptake of [^18^F]2 is indicative of defluorination

The in vivo biodistribution of **[**^**18**^**F]2** was evaluated in C57BL/6 female mice receiving a dose of gonadotropins (15 IU FSH + 15 IU LH) plus 5 IU hCG, 48 and 6 h to induce PR expression in the ovary; PR was not induced in the control mice (Figs. 4 and 5). The upregulation of PR was confirmed by ex vivo western blot analysis after the imaging study (Fig. 3a). Biodistribution analysis at 50 min showed a non-significant increase in uptake of **[**^**18**^**F]2** in the ovary of the control animals (1.32 ± 0.22 %ID/g) and GH stimulated animals (2.17 ± 0.03 %ID/g); however, more notable was the large accumulation of radioactivity in bone and small intestine in both cohorts of mice.

### Metabolite analysis

**Fig. 6:**
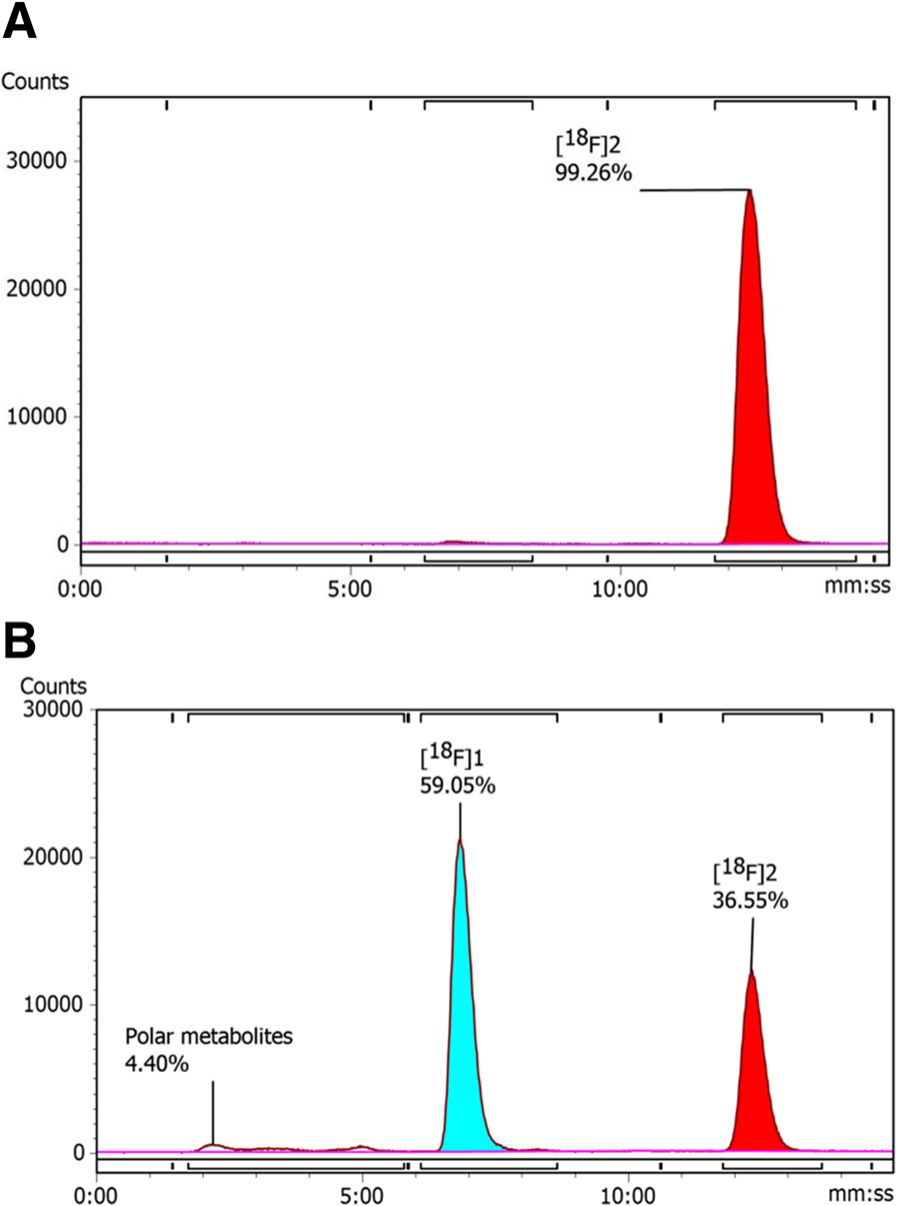
Representative analytical RP-HPLC chromatograms of: **a** parent compound [^18^F]2; **b** analysis after incubation of **[**^**18**^**F]2** with MLM for 60 min

The rapid in vivo defluorination of **[**^**18**^**F]2** was surprising as similar 2-fluoropyridine compounds are reported to be metabolically stable; to evaluate this further in vitro metabolite analysis using mouse liver microsomes (MLM) was performed to inform future radioligand development.(Bouvet et al., 2016, Mccarron et al., 2004) Polar metabolites were observed after incubating **[**^**18**^**F]2** with MLM for 60 min (4.4%, R_t_ = 2:00–5:00 min:sec) however, the major metabolite (63 ± 1.9%) was a single peak at *ca* t_R_ = 7:05 (Fig. 6). The retention time of this metabolite corresponded with that of compound **1**, the oxocarbamate derivative of **2**, therefore it was speculated that oxidative metabolism converted **[**^**18**^**F]2** into **[**^**18**^**F]1**. This metabolic pathway has been previously described for Tanaproget and the conversion may be facilitated through an S-linked glucuronide intermediate (Keating et al., 2006). The conversion was confirmed by repeating the experiment and spiking the parent **[**^**18**^**F]2** with an aliquot of **[**^**18**^**F]1** (Additional file 1: Figure S36). Spectral analysis of the metabolite was confounded by the low mass of material required to obey the tracer dose principle; therefore, the experiment was repeated on a macroscopic scale by incubating compound **2** with MLM and human liver microsomes (HLM). Metabolites were identified by mass spectrometry and drug clearance was evaluated. Metabolite identification confirmed the conversion of the thiocarbamate into oxocarbamate as the major metabolic pathway and highlighted oxidative defluorination of **2** in both MLM and HLM (Additional file 1: Tables S5 and S6). Unfortunately, the exact structure of the oxidised and defluorinated metabolite could not be resolved from the mass spectra although we propose that an initial oxidation may facilitate the defluorination. Compound **2** was cleared rapidly from MLM at a rate of 1270 μL/min/mg protein and ca. 22-fold slower in HLM (56 μL/min/mg protein), suggesting that the rapid metabolism of **2** may be species dependent; however, defluorination was evident in both MLM and HLM, therefore it is unlikely that the fate of **[**^**18**^**F]2** would be different in humans, especially at the sub-nanomolar quantities of **[**^**18**^**F]2** present in a tracer dose. We also performed in vitro metabolite analysis of compound **1** in MLM and HLM to determine if defluorination was independent of the presence of the thiocarbamate; compound **1** showed defluorinated species in both MLM and HLM experiments with a comparable clearance. The presence of a defluorinated metabolite in MLM corroborates the rapid in vivo defluorination of **[**^**18**^**F]2**.

## Discussion

The synthesis of a focused library of PR ligands was guided by the SAR of Tanaproget and other benzoxazin(thi)one derivatives. Five design criteria were proposed to structure the synthetic strategy which yielded the focused library described in Fig. 2:

i) The ligand must contain a fluoro-aryl substituent to facilitate fluorine-18 radiochemistry;
ii) Structural diversity through derivatisation of the 6-aryl position;
iii) Preference for achiral compounds to avoid requirement for chiral purity during radiochemistry steps and formulation;
iv) Ligands may contain a cyclic carbamate or thiocarbamate;
v) Ligands should exhibit a cLogP < 5 to limit non-specific binding.

Modifying the substituent at the 6-aryl position created a library of structurally diverse, fluorine-containing compounds. For each compound, the oxocarbamate and thiocarbamate derivative was synthesised to evaluate the effect of carbonyl and thiocarbonyl moieties on potency. An advantage of our synthetic methodology was the potential for intermediates **2b**, **4b** and **6b** to be radiolabelled with carbon-11 using a [^11^C]CS_2_ methodology previously reported for the radiosynthesis of [^11^C]Tanaproget (Haywood et al., 2015).

In the potency assay comparison between oxo- and thiocarbamate compounds showed the “flip” between agonist and antagonist profiles in agreement with the literature (Table 1) (Fensome et al., 2005). Compounds **1** and **3** were predicted to be the least lipophilic compounds in the library; however they did not induce an agonist response within the assay parameters (EC_50_ < 10,000 nM), suggesting a stronger antagonist profile. The ability to bind PR was confirmed by competition with progesterone (3 mM); **1** and **3** exhibited low micromolar antagonist activity (844.8 ± 0.23 and 795.0 ± 0.25 nM respectively). Compound **6** was *ca* 5-fold more potent (EC_50_ = 432.5 ± 0.24 nM) than the corresponding oxocarbamate derivative **5** (EC_50_ = 2294 ± 0.29); with cLogP values ≥5 and low micromolar potency, these compounds did not meet the required characteristics for evaluation as PR imaging agents**.** The most potent compound in the library was **2** (EC_50_ = 4.7 ± 0.07 nM) and was predicted to exhibit a similar lipophilicity to Tanaproget. The binding specificity of compound **2** was evaluated in a GR nuclear translocation assay using CHO-K1 cells and failed to elicit a response at 0.5 μM, suggesting little cross-reactivity (Additional file 1: Figure S33). The failure to elicit a response in the GR nuclear translocation assay provided confidence that **2** was specific to PR without evaluating the full SHR family. Confidence in extrapolating cross-reactivity to the whole SHR family based upon GR alone came from the high degree of homology between SHRs and the extensive investigation into the cross-reactivity of benzoxazin(thi)one derivatives in the literature.

**Fig. 7:**
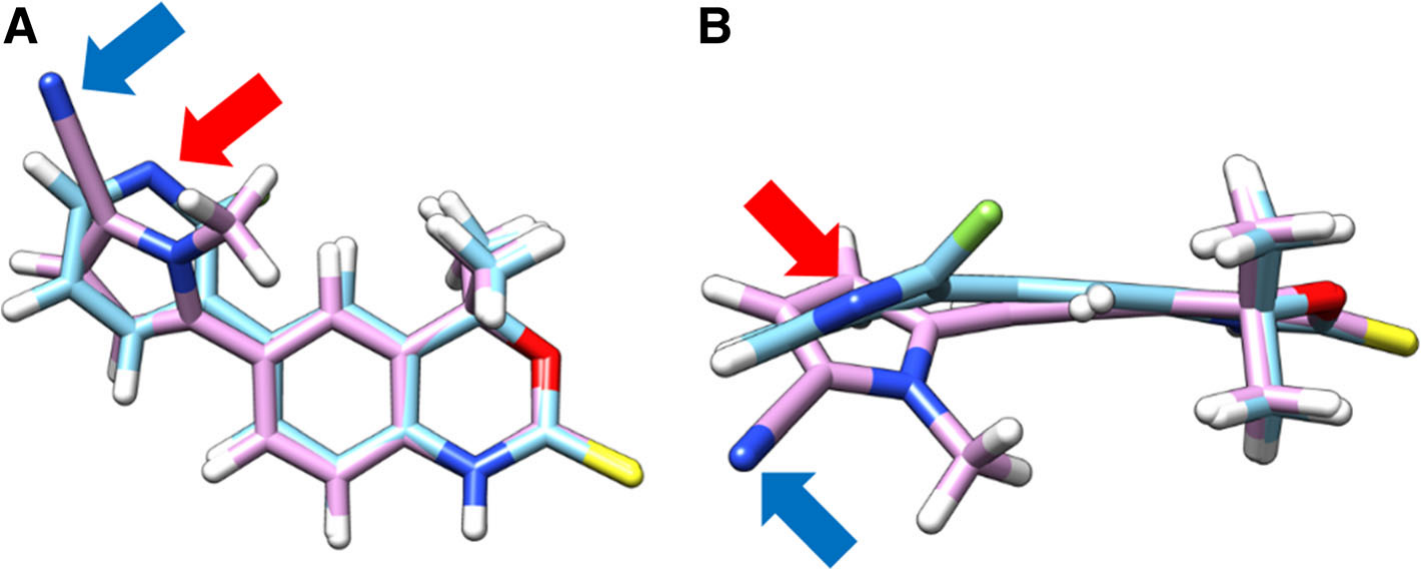
A three-dimensional representation of Tanaproget and compound **2**, overlaid at the benzoxazinthione pharmacophore: **a** side-view; **b** top-down view; red arrow: nitrogen of the pyridine moiety of **2**; blue arrow: nitrogen of the nitrile moiety of Tanaproget

It is not clear why **2** exhibited greater potency compared to structural isomer **4** (*ca* 1000-fold more potent). The crystal structure of Tanaproget bound to PR demonstrates that the nitrile group forms important hydrogen bonds with the Gln725 and Arg766 amino acid residues (distance of 3 and 2.6 Å respectively) (Zhang et al., 2005). The pyridine moiety of **2** and **4** is also a potential hydrogen bond acceptor with one (or both) of the residues. Three-dimensional models of Tanaproget and **2** were overlaid and aligned at the benzoxazinthione pharmacophore, the aryl moiety in the C6-position of both molecules was rotated and brought the nitrile of Tanaproget (Fig. 7, blue arrow) and the nitrogen of the pyridine (Fig. 7, red arrow) in compound **2** into close proximity in space providing tentative evidence that **2** may be capable of forming hydrogen bonds with the same residues as Tanaproget. We propose that the 6-fluoro substitution in compound **4** sterically forces the molecule to adopt a binding pose in the LBD that is unfavourable for hydrogen bonding. The 2-fluoro substitution of compound **2** does not perturb a favourable binding pose and the formation of hydrogen bonds, which accounted for the low nanomolar potency of **2**.

Compound **2** was radiolabelled using nucleophilic heteroaromatic substitution (S_N_Ar) at the 2-position of the pyridine ring by displacement of a nitro leaving group without the necessity of an electron-withdrawing substituent (Scheme 2) (Dolle, 2005). Furthermore, the 2-fluoropyridin-3-yl moiety in compound **2** presented the opportunity to utilise well-established S_N_Ar radiochemistry to access an intermediate radiolabelled pyridine. Consistent with the radiosynthesis of [^18^F]FPTP and our preliminary studies, direct radiolabelling of a thiocarbamate precursor did not yield **[**^**18**^**F]2**, likely due to the increased nucleophilicity of the thiocarbonyl compared to oxocarbonyl (data not shown). Conversion to the thiocarbamate was required post-radiolabelling as exemplified in the radiosynthesis of [^18^F]FPTP (Lee et al., 2010).

Biodistribution in hormone stimulated female mice (Fig. 4) showed uptake of radioactivity in the bone was 2-fold higher in the GH stimulated mice (31.21 ± 5.99 %ID/g) compared to the control group (15.36 ± 3.50 %ID/g); growth hormones are known to induce the expression of metabolic enzymes in the liver, therefore an increase in radioactivity uptake in the bone is suggestive of metabolic defluorination of **[**^**18**^**F]2** (Waxman and Holloway, 2009). PET images acquired over 50 min showed an accumulation of radioactivity in the liver and small intestines, but also in the spine, joints and skull (Fig. 5 and movie in ESI). Time activity curves acquired over the dynamic scan showed rapid accumulation of radioactivity in the small intestine with an increase in bone uptake over time, indicative of radioligand defluorination (Additional file 1: Figure S34). The poor in vivo stability of **[**^**18**^**F]2** did not warrant further investigation into alternative preclinical animal models. The metabolic oxidation of the benzoxazinthione pharmacophore to the corresponding oxocarbamate has not been reported previously in the context of radioligand development and represents an important finding for the future development of PET radioligands derived from this pharmacophore. Furthermore, we present an example of a metabolically unstable 2-[^18^F]fluoropyridine moiety, previously assumed to be resistant to in vivo defluorination based upon the current literature.

## Conclusions

The synthesis and biological evaluation of a focused library of non-steroidal PR ligands for PET imaging highlighted compound **2** as a candidate for further biological evaluation. A radiosynthetic route to access **[**^**18**^**F]2** was developed to allow in vitro and in vivo evaluation. Radioligand **[**^**18**^**F]2** demonstrated significant in vitro uptake in PR++ T47D cells which could be blocked in a dose dependent manner with progesterone. However, **[**^**18**^**F]2** showed poor in vivo metabolic stability with rapid defluorination within the time frame of the imaging protocol. In vitro metabolite analysis of **2** in MLM confirmed defluorination and oxidative metabolism of the thiocarbamate to oxocarbamate moiety by mass spectrometry. In summary, **[**^**18**^**F]2** has inadequate stability for in vivo imaging in mice and with a similar metabolic profile in HLM, is predicted to be unsuitable for use in humans. The future development of PR imaging agents based around the benzoxazinthione pharmacophore should proceed with caution due to the described metabolic conversion into the benzoxazinone derivative. In addition, we have shown that 2-[^18^F]fluoropyridine moieties should not be assumed to be metabolically stable to defluorination. In a scenario where **[**^**18**^**F]2** was stable to defluorination, conversion from the thiocarbamate into the oxocarbamate may change the properties of the ligand such as receptor affinity which could confound in vivo evaluation. The development of metabolically stable non-steroidal imaging agents targeting PR is ongoing.

## Additional file

Additional file 1: Reaction Scheme for synthesis of precursor 7; ^1^H, ^13^C, ^19^F NMR data and HPLC purity analysis for compounds 1, 2, 3, 4, 5, 6 and 7; radio-HPLC analysis of [^18^F]1 and [^18^F]2; Molar activity calibration curve; Log D calculation raw data; plotted data for T47D assay; In vitro cross reactivity assay data; TACs for in vivo evaulation of [^18^F]2; ex vivo biodistribution data for [^18^F]2; HPLC metabolite analysis for [^18/19^F]2; MS metabolite analysis for [^19^F]2. (DOCX 3180 kb)

## Abbreviations

[^18^F]FES: 16α-[^18^F]Fluoro-17β-estradiol
[^18^F]FFNP: 21-[^18^F]Fluorofuranyl-norprogesterone
[^18^F]FMTP: [^18^F]Fluoromethyl-tanaproget
[^18^F]FPTP: [^18^F]Fluoropropyl-tanaproget
20-HSD: 20α-hydroxysteroid dehydrogenase
AR: Androgen receptor
CPM: Counts per minute
ER: Estrogen receptor
GR: Glucorticoid receptor
HLM: Human liver microsomes
LBD: Ligand binding domain
LI: Large intestine
MDR: Multi-drug resistance
MLM: Mouse liver microsomes
PBS: Phosphate buffered saline
PET: Positron emission tomography
PR: Progesterone receptor
RBA: Relative binding affinity
RCP: Radiochemical purity
RP-HPLC: Reverse-phase high performance liquid chromatography
RPMI: Roswell Park Memorial Institute
SAR: Structure activity relationship
SD: Standard deviation
SEM: Standard error of the mean
SERM: Selective estrogen receptor modulator
SHR: Steroid hormone receptor
SI: Small intestine
SPE: Solid phase extraction
TCDI: 1,1’-Thiocarbonyldiimidazole
