# Supplementary material for: Synthesis of a benzoxazinthione derivative of tanaproget and pharmacological evaluation for PET imaging of PR expression
Source: EJNMMI Radiopharm Chem. 2019 Jan 10;4:1. doi: 10.1186/s41181-018-0054-z (PMC6328429; doi:10.1186/s41181-018-0054-z)

**Additional file 1**

**Synthesis of a benzoxazinthione derivative of Tanaproget and pharmacological evaluation for PET imaging of PR expression**

**Authors:**Louis Allott‡ ^1^, Cecilia Miranda‡ ^2^, Angela Hayes^3^, Florence Raynaud^3^, Christopher Cawthorne *^2,4^, Graham Smith*^1^.

^1^ Division of Radiotherapy and Imaging, The Institute of Cancer Research, 123 Old Brompton Road, London, U.K.
^2^ PET Research Centre, University of Hull, Cottingham Road, Hull, Yorkshire, HU6 7RX, U.K.
^3^ Cancer Therapeutics, The Institute of Cancer Research, 123 Old Brompton Road, London, U.K.

^4^ Nuclear Medicine and Molecular Imaging, Department of Imaging and Pathology/MoSAIC- Molecular Small Animal Imaging Centre, KU Leuven, Belgium

*Corresponding authors:

Graham Smith, Tel: +44 20 8722 4482, Fax: +44 20 7370 5261, Email address: graham.smith@icr.ac.uk
Christopher Cawthorne, Tel: +32 16325694, Email address: christopher.cawthorne@kuleuven.be

‡ Authors contributed equally.

**Contents:**1. Materials and Methods Page 02
2. Precursor synthetic scheme Page 02
3. NMR spectra Page 03
4. Purity RP-HPLC chromatograms Page 13
5. Radiochemistry RP-HPLC chromatograms Page 16
6. Molar activity calibration curve Page 19
7. Distribution coefficient analysis Page 20
8. *In vitro* T47D AP assay Page 21
9. *In vitro* Cross-reactivity assay Page 22
10. *In vivo evaluation* Page 23
11. In vitro metabolism of [^18^F]2 Page 25
12. Mass spectrometry metabolite identification Page 28

**1. Materials and Methods**

^1^H, ^13^C and ^19^F NMR spectra were obtained using a Bruker 500 MHz spectrometer operating at room temperature. Chemical shifts (δ) are reported in parts per million (ppm) and residual solvent peaks have been used as an internal reference. Peak multiplicities have been abbreviated as follows: s (singlet), d (doublet), dd (double-doublet), m (multiplet). Compound purity, radiochemical semi-preparative RP-HPLC and radiochemistry quality control was determined by RP-HPLC using an Agilent Infinity 1260 quaternary pump system equipped with a 1260 diode array (Agilent Technologies). Elution profiles were analysed using Laura software (Lablogic, Sheffield, UK), The purity of compounds **1** – **7** were determined using a Zorbax Eclipse XDB C18 column, 4.6 x 150 mm, 5 µm (Agilent Technologies) with gradient 1: **0-20** min 3%-90% B a flow rate of 1 mL/min with 0.1% TFA in water as eluent A and 0.1% TFA in MeCN as eluent B. Retention times (R_t_) are expressed as minutes:seconds (mm:ss).

**2. Precursor synthetic scheme**

**Scheme S1.** Synthesis of a radiochemistry precursor. Reaction conditions: Pd(PPh_3_)_2_Cl­_2_, K_2_CO_2_, MeCN:H_2_O (1:1), µW 150 °C, 200W, 20 min.

A 2-nitropyridine precursor (**7**) was synthesised following Scheme S1 to access radiolabelled compound **[^18^F]1** which was converted into **[^18^F]2** post-radiolabelling.

**3. NMR Spectra**

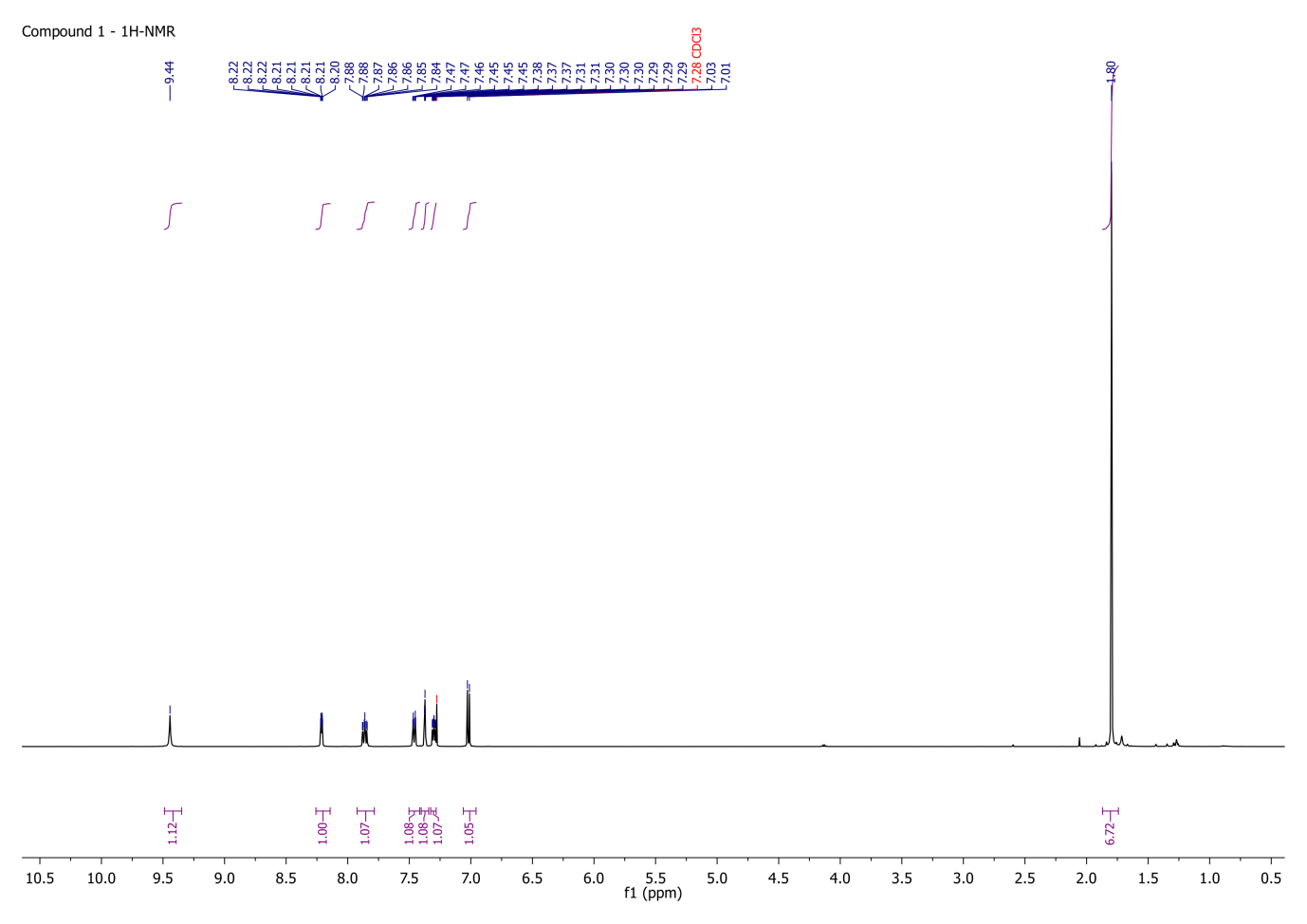
**Figure S1.** ^1^H-NMR of *6-(2-fluoropyridin-3-yl)-4,4-dimethyl-1H-benzo[d][1,3]oxazin-2(4H)-one (****1****)*


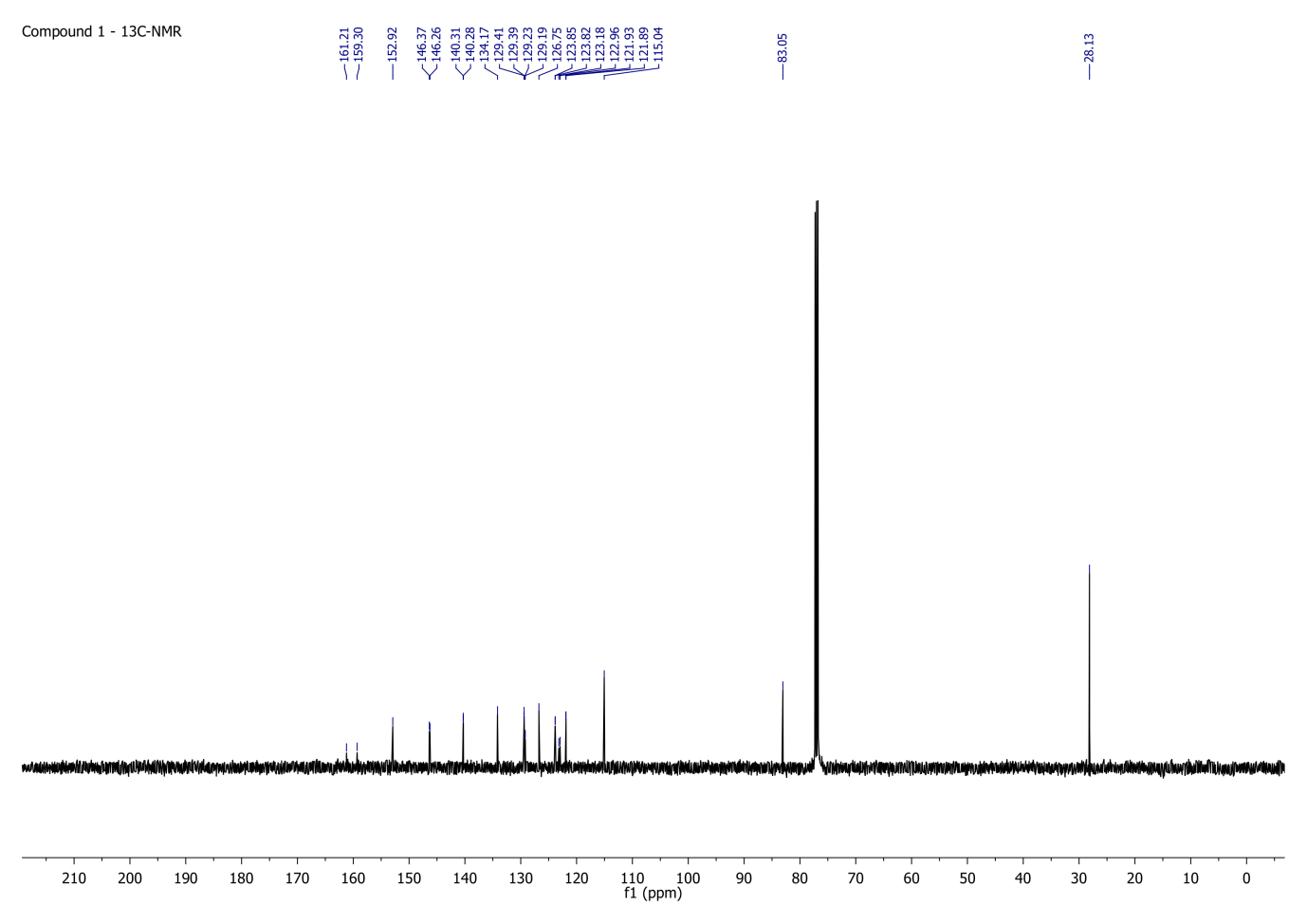
**Figure S2.** ^13^C-NMR of *6-(2-fluoropyridin-3-yl)-4,4-dimethyl-1H-benzo[d][1,3]oxazin-2(4H)-one (****1****)*

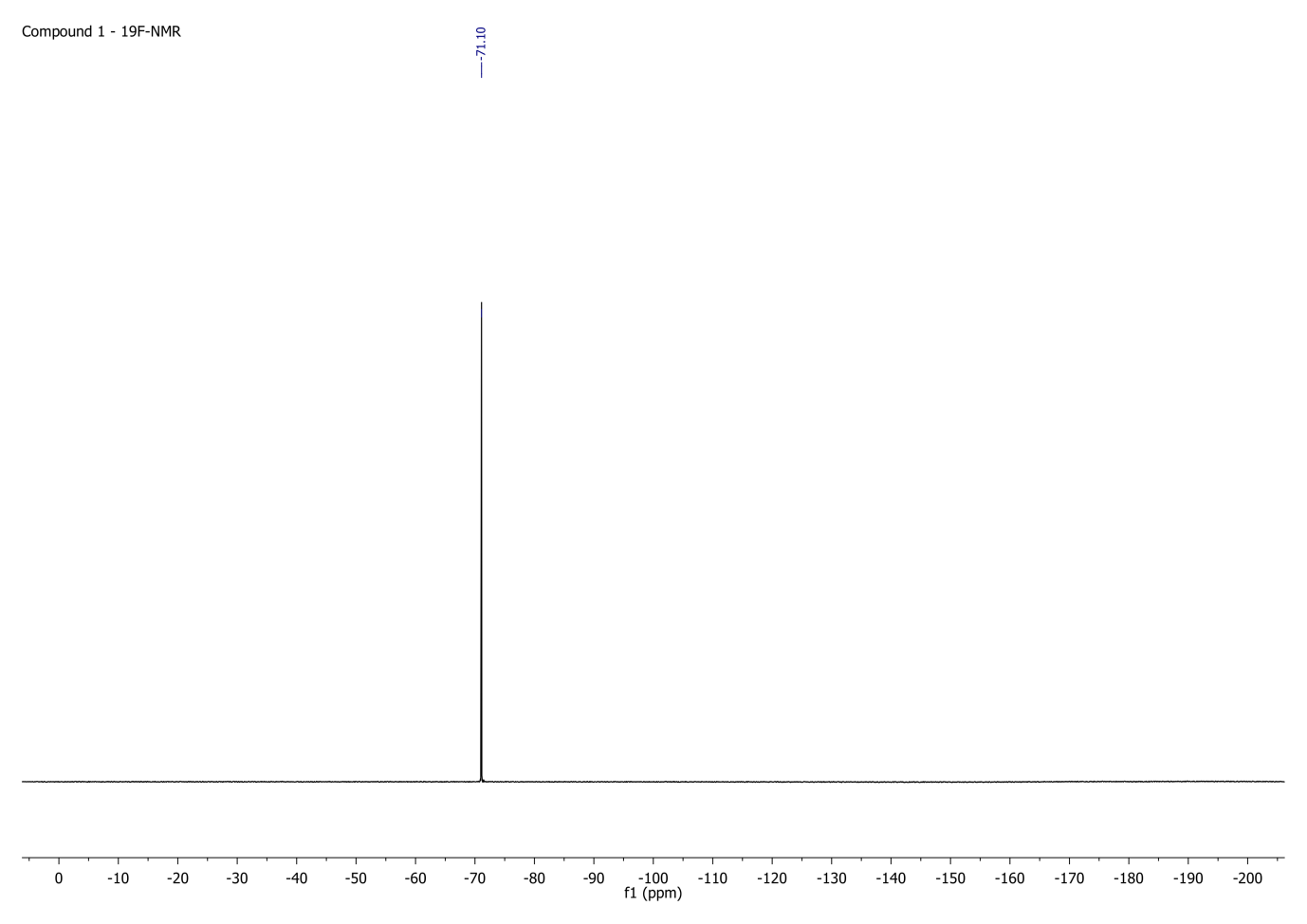

**Figure S3.** ^19^F-NMR of *6-(2-fluoropyridin-3-yl)-4,4-dimethyl-1H-benzo[d][1,3]oxazin-2(4H)-one (****1****)*


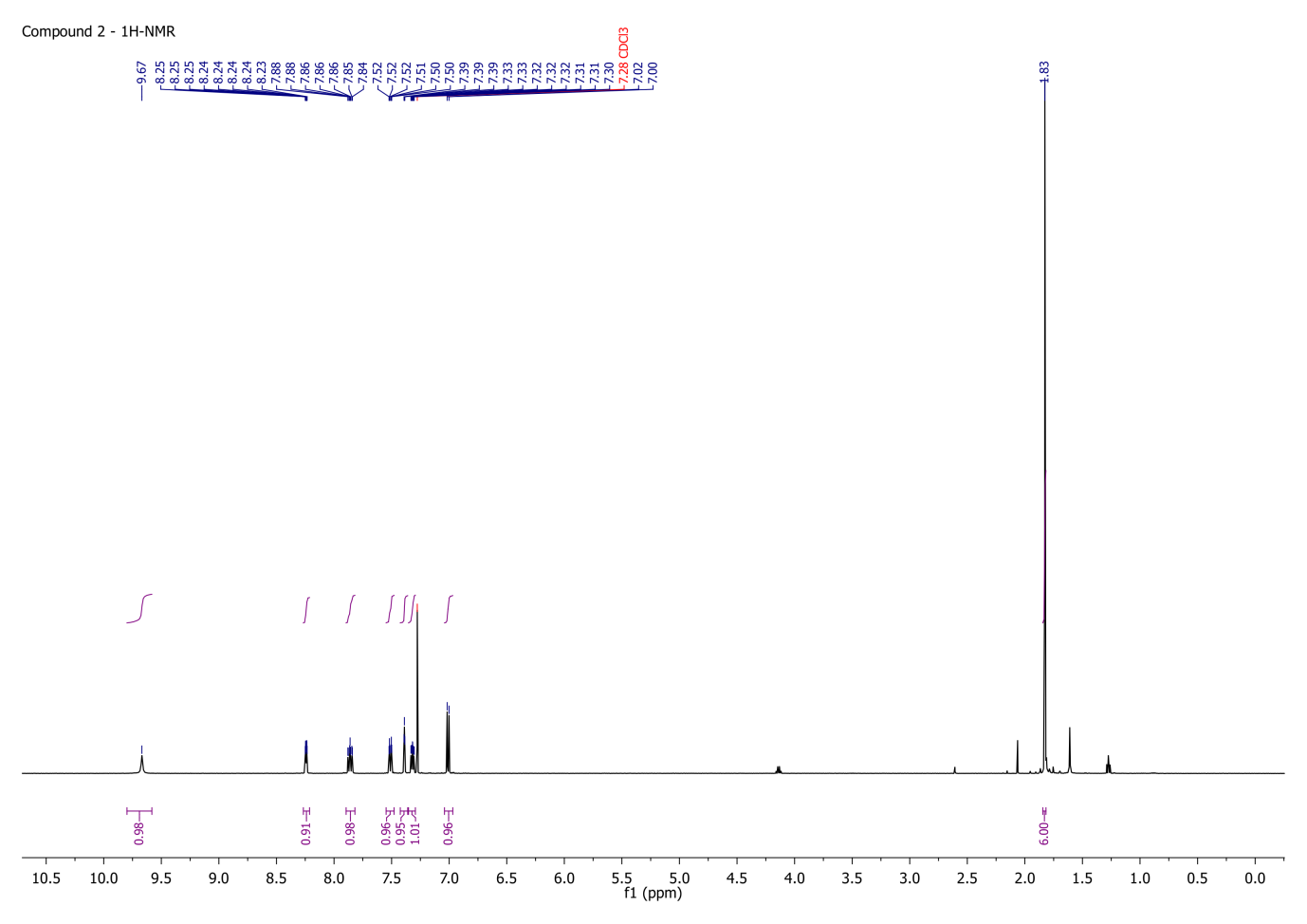

**Figure S4.** ^1^H-NMR of *6-(2-fluoropyridin-3-yl)-4,4-dimethyl-1H-benzo[d][1,3]oxazine-2(4H)-thione (****2****).*


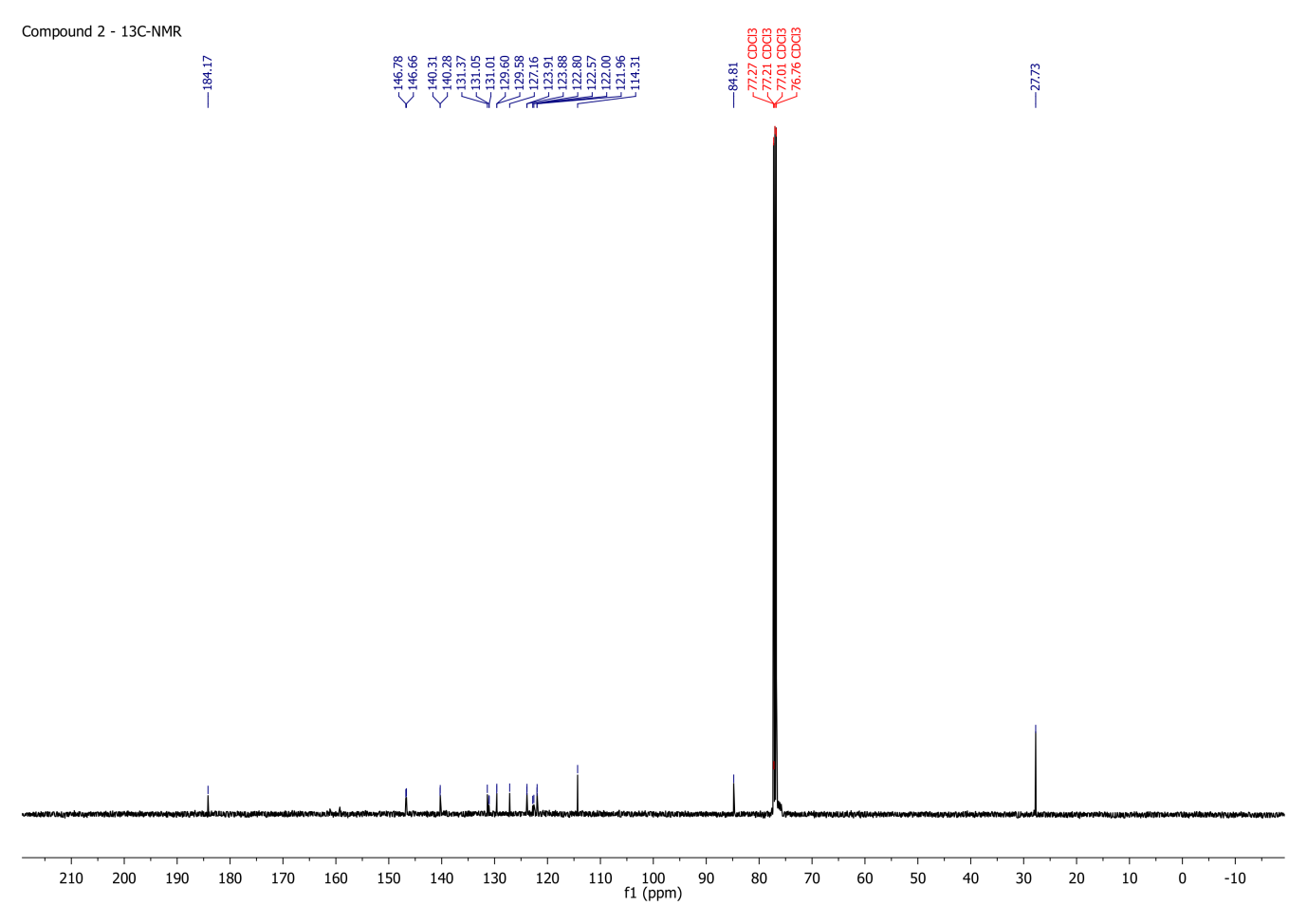

**Figure S5.** ^13^C-NMR of *6-(2-fluoropyridin-3-yl)-4,4-dimethyl-1H-benzo[d][1,3]oxazine-2(4H)-thione (****2****).*


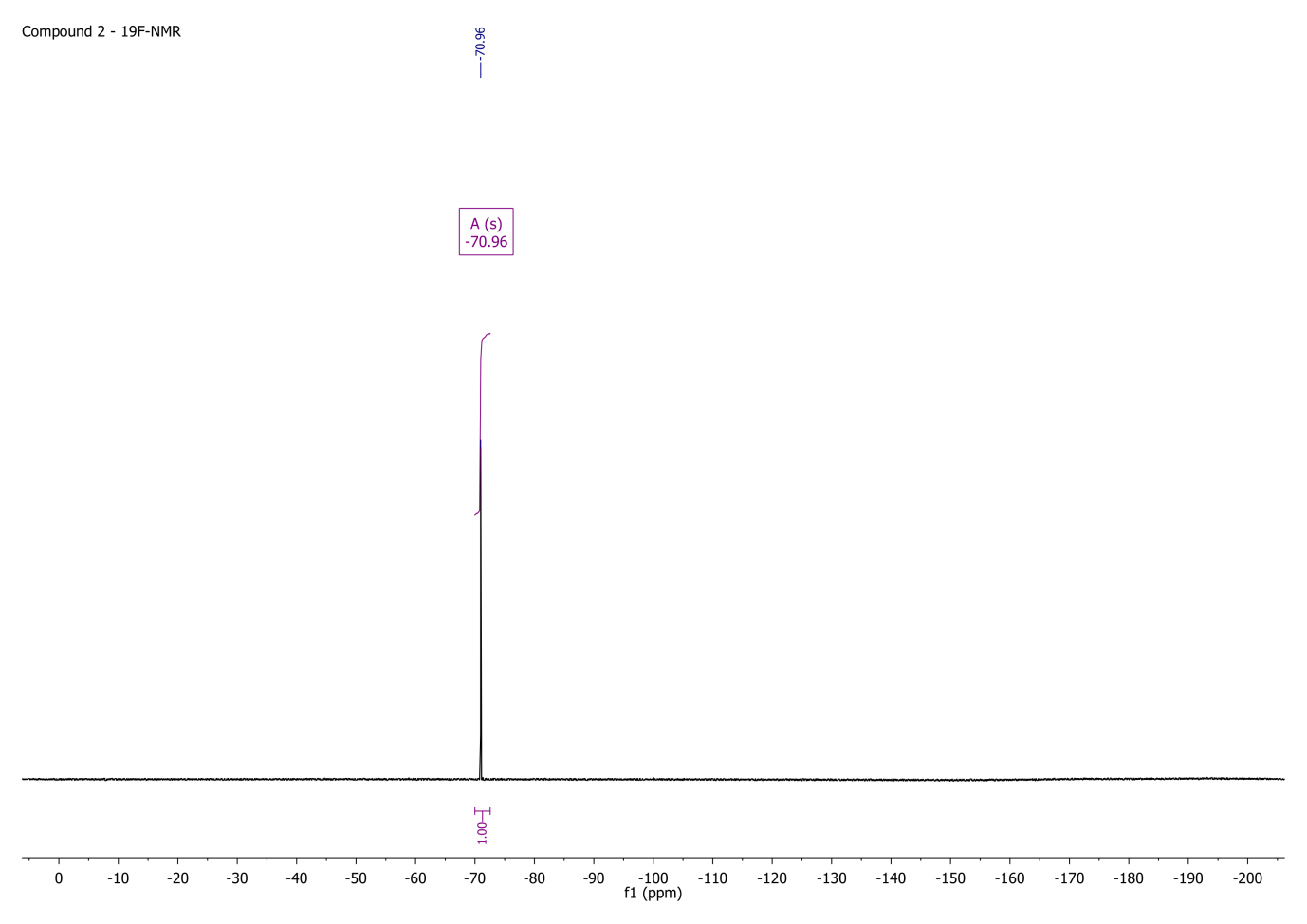

**Figure S6.** ^19^F-NMR of *6-(2-fluoropyridin-3-yl)-4,4-dimethyl-1H-benzo[d][1,3]oxazine-2(4H)-thione (****2****).*

*
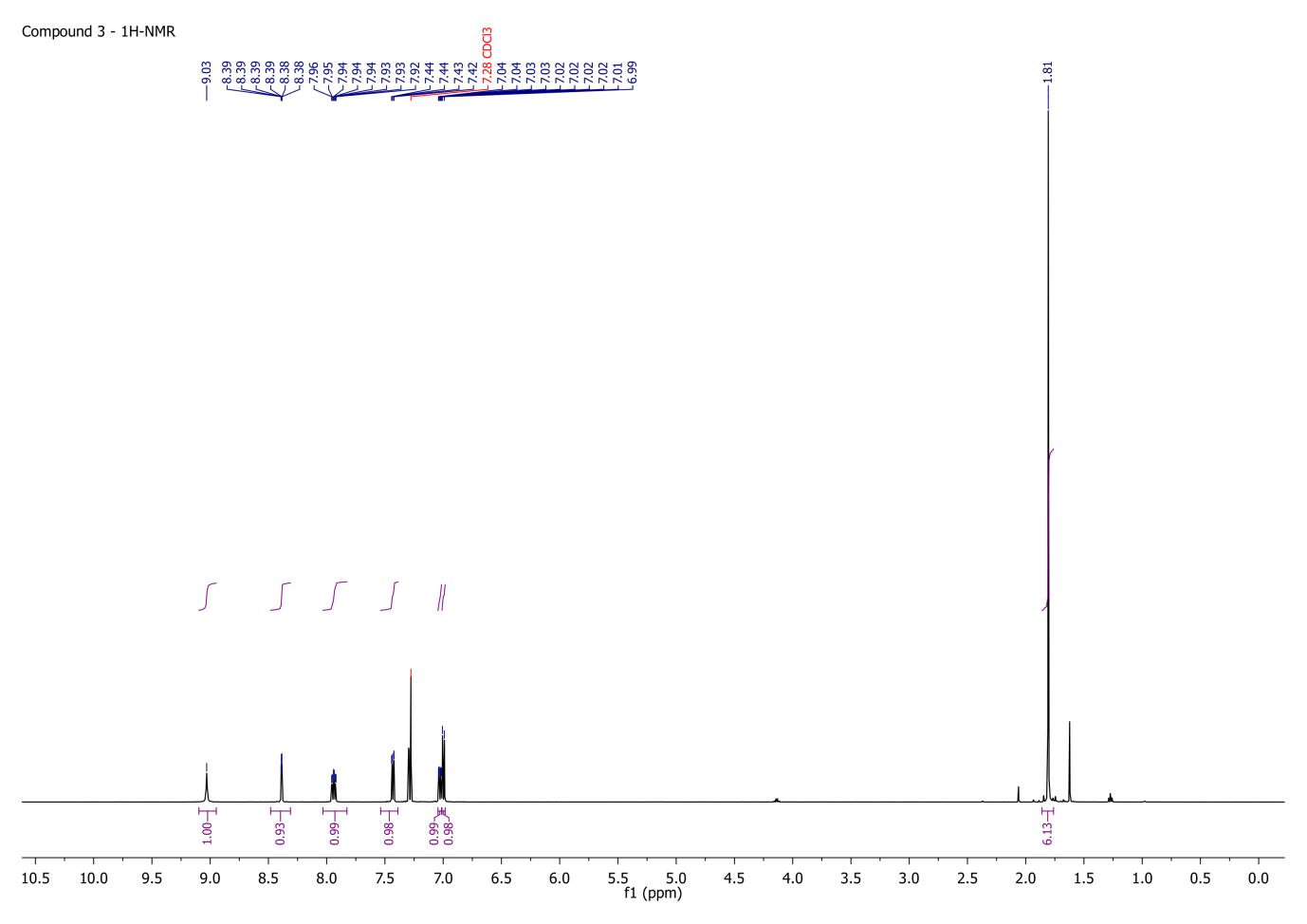
*

**Figure S7.** ^1^H-NMR of *6-(6-fluoropyridin-3-yl)-4,4-dimethyl-1H-benzo[d][1,3]oxazin-2(4H)-one (****3****).*


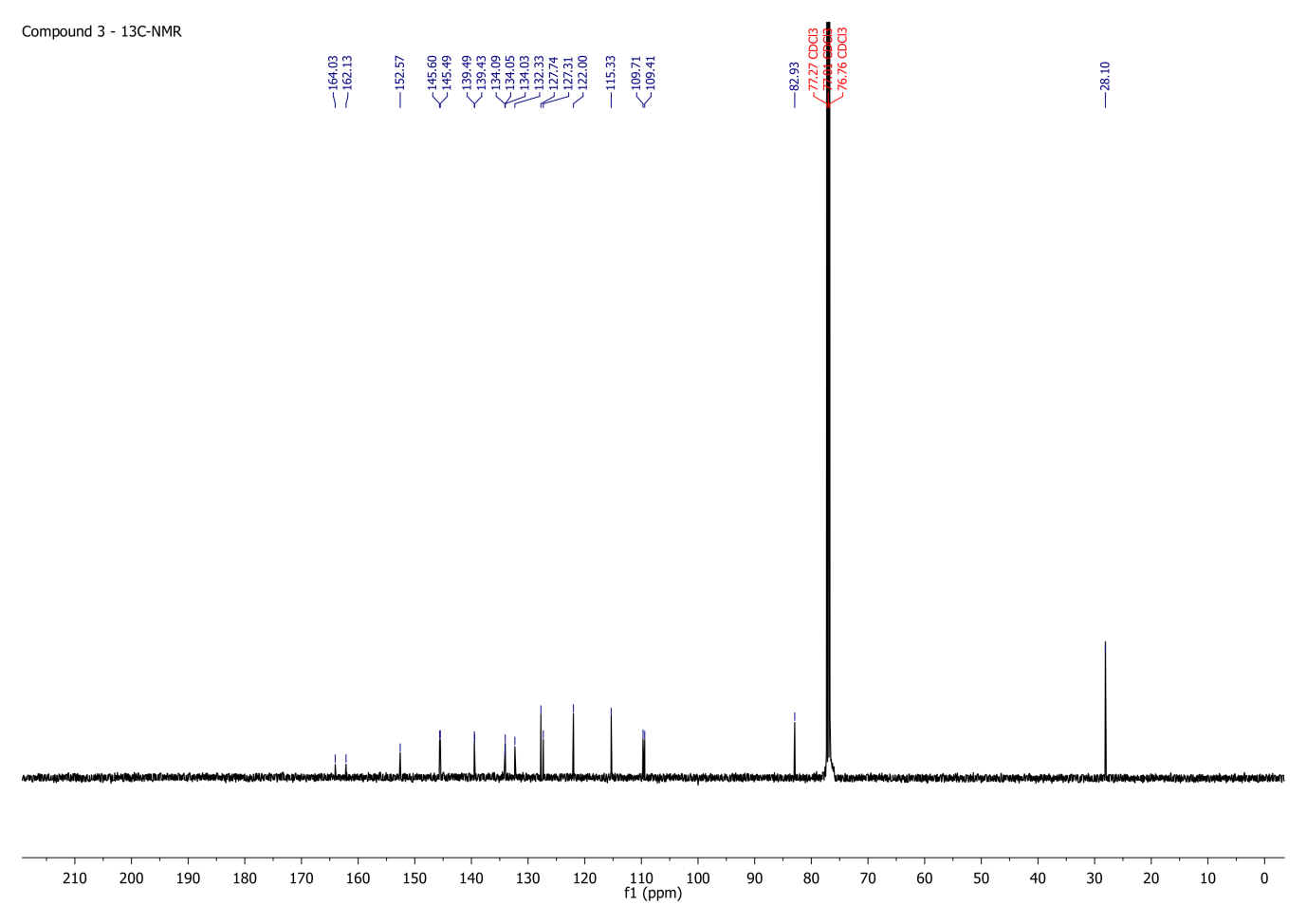

**Figure S8.** ^13^C-NMR of *6-(6-fluoropyridin-3-yl)-4,4-dimethyl-1H-benzo[d][1,3]oxazin-2(4H)-one (****3****).*


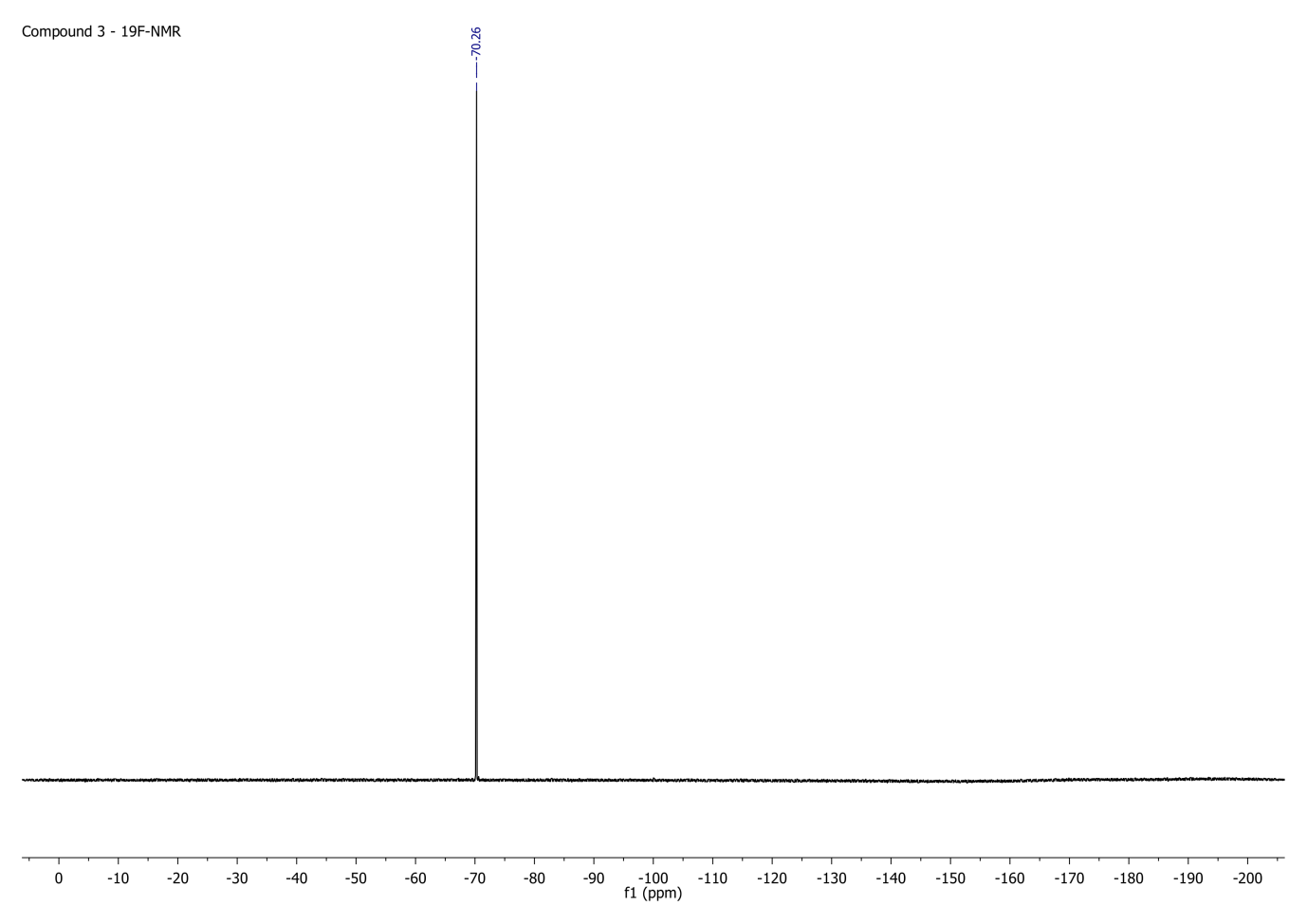

**Figure S9.** ^19^F-NMR of *6-(6-fluoropyridin-3-yl)-4,4-dimethyl-1H-benzo[d][1,3]oxazin-2(4H)-one (****3****).*


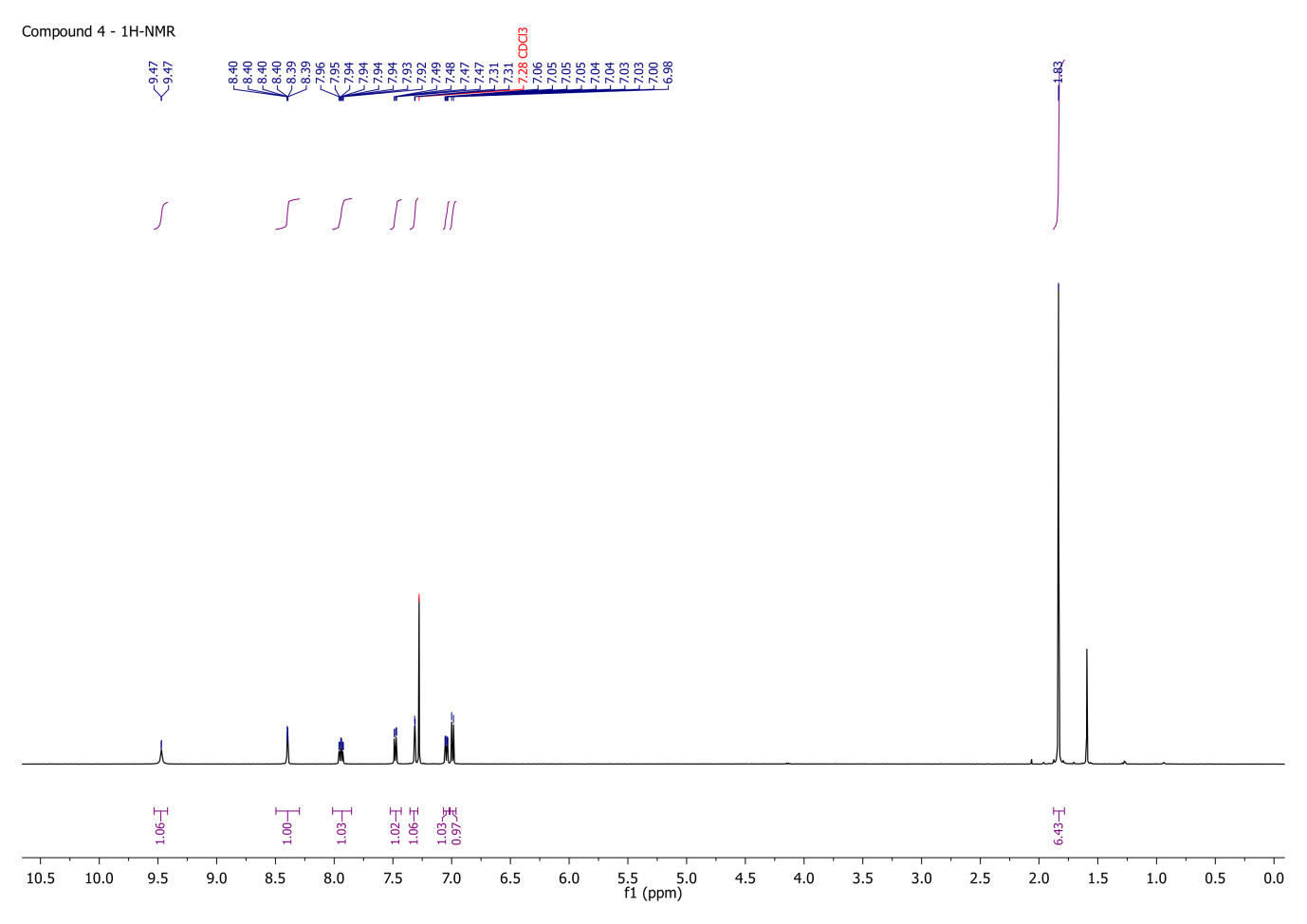

**Figure S10.** ^1^H-NMR of *6-(6-fluoropyridin-3-yl)-4,4-dimethyl-1H-benzo[d][1,3]oxazine-2(4H)-thione (****4****).*

*
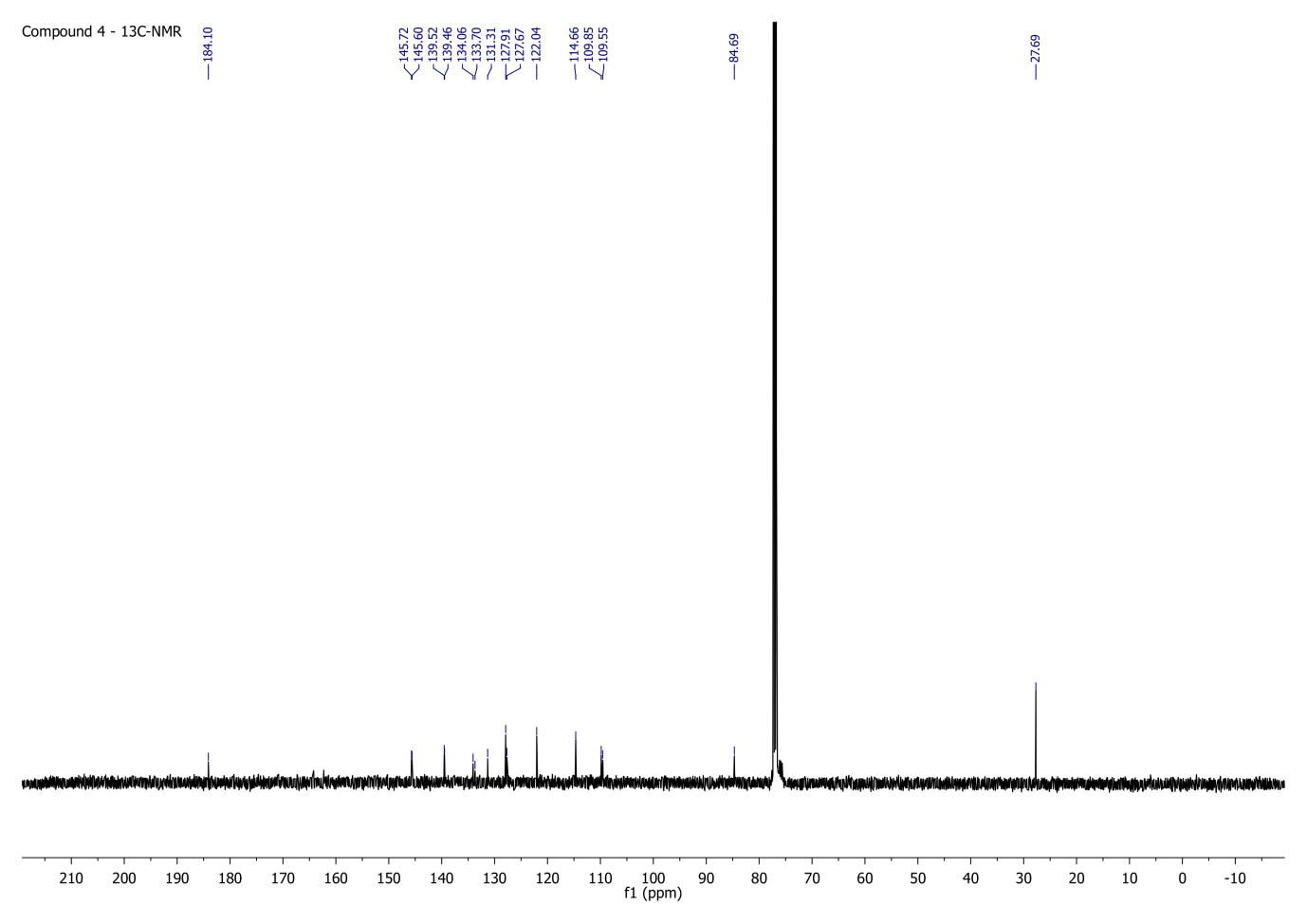
*

**Figure S11.** ^13^C-NMR of *6-(6-fluoropyridin-3-yl)-4,4-dimethyl-1H-benzo[d][1,3]oxazine-2(4H)-thione (****4****).*

*
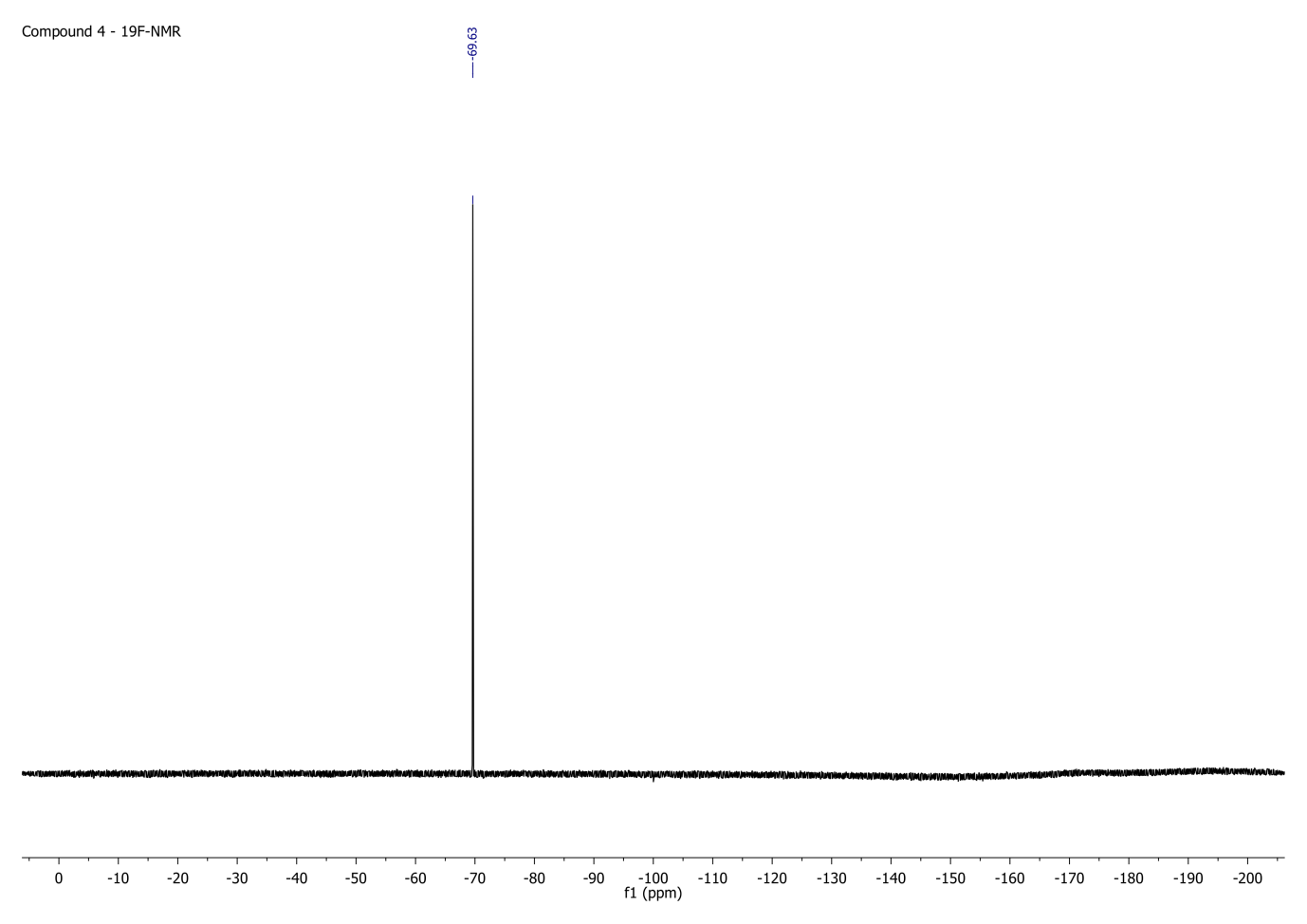
*

**Figure S12.** ^19^F-NMR of *6-(6-fluoropyridin-3-yl)-4,4-dimethyl-1H-benzo[d][1,3]oxazine-2(4H)-thione (****4****).*


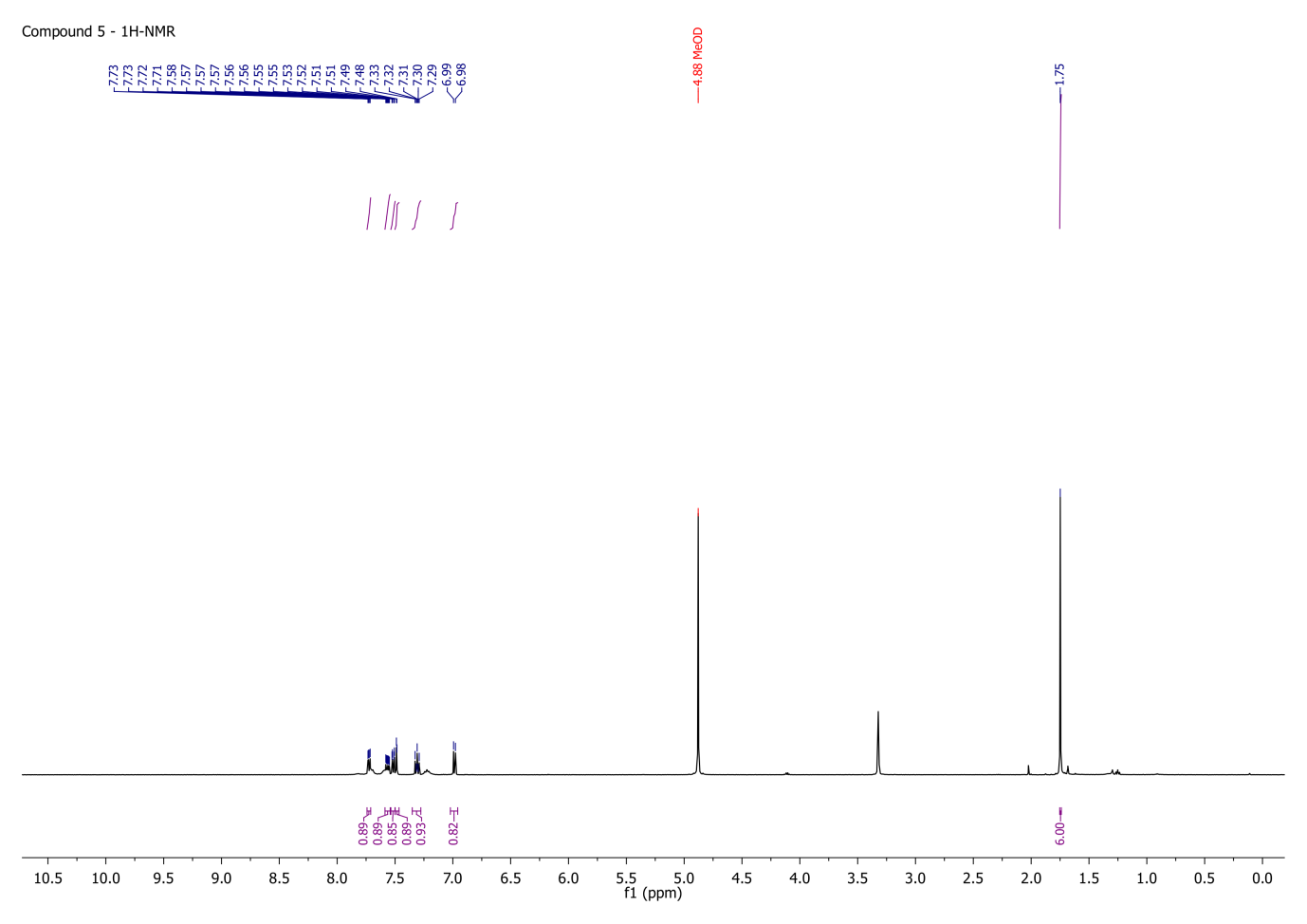
**Figure S13.** ^1^H-NMR of *6-(3-chloro-4-fluorophenyl)-4,4-dimethyl-1H-benzo[d][1,3]oxazin-2(4H)-one* *(****5****).*

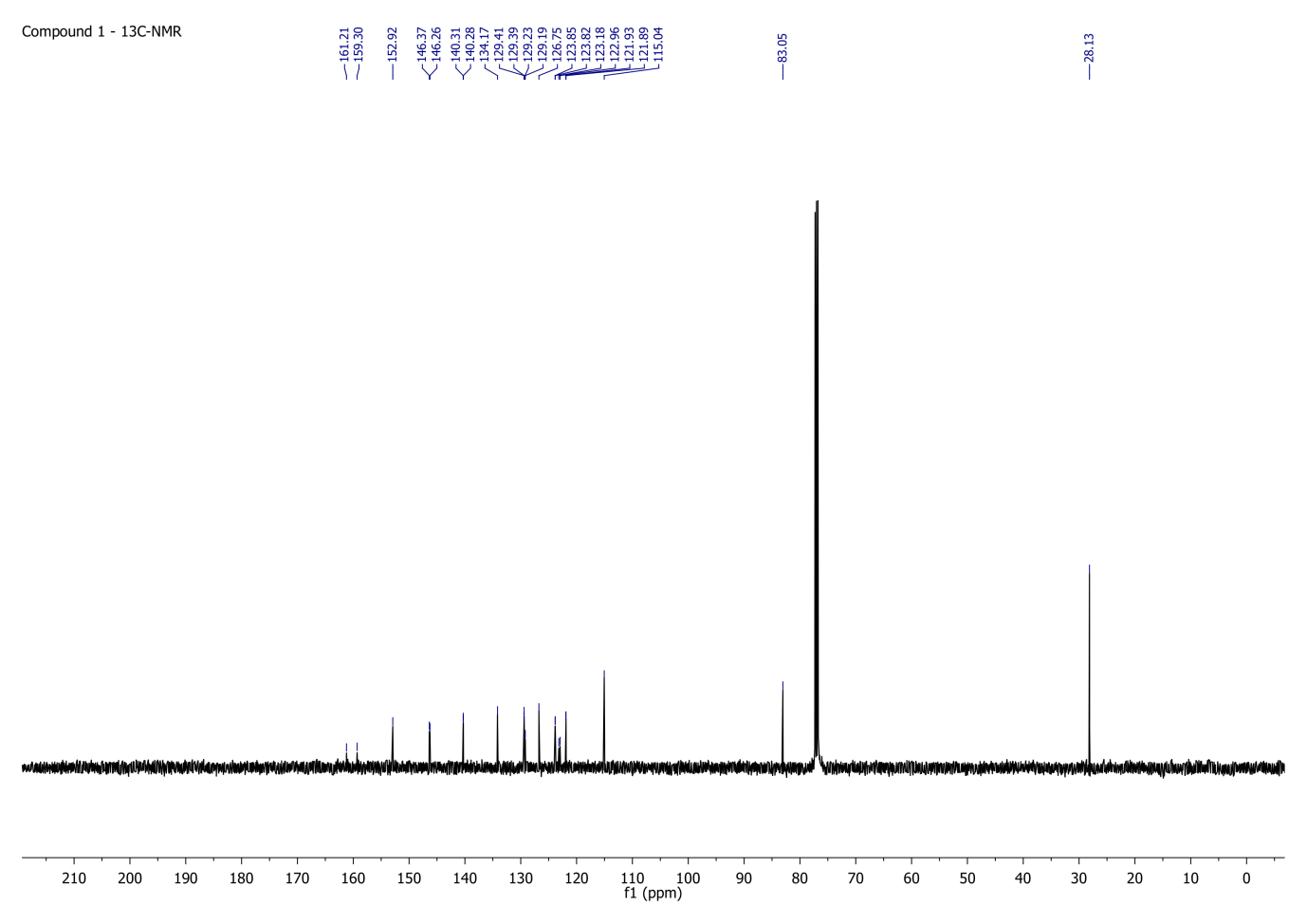

**Figure S14.** ^13^C-NMR of *6-(3-chloro-4-fluorophenyl)-4,4-dimethyl-1H-benzo[d][1,3]oxazin-2(4H)-one* *(****5****).*


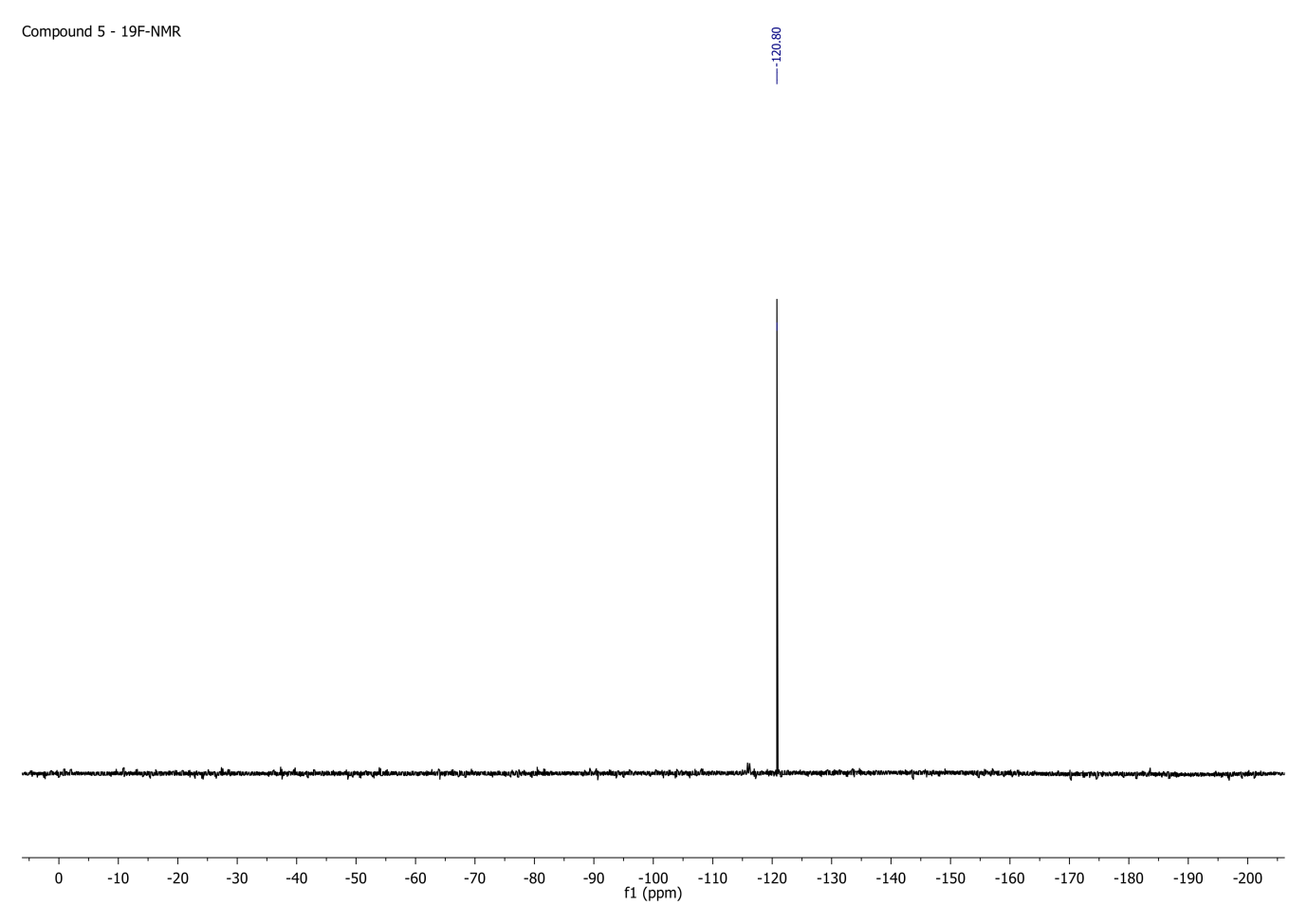

**Figure S15.** ^19^F-NMR of *6-(3-chloro-4-fluorophenyl)-4,4-dimethyl-1H-benzo[d][1,3]oxazin-2(4H)-one* *(****5****).*


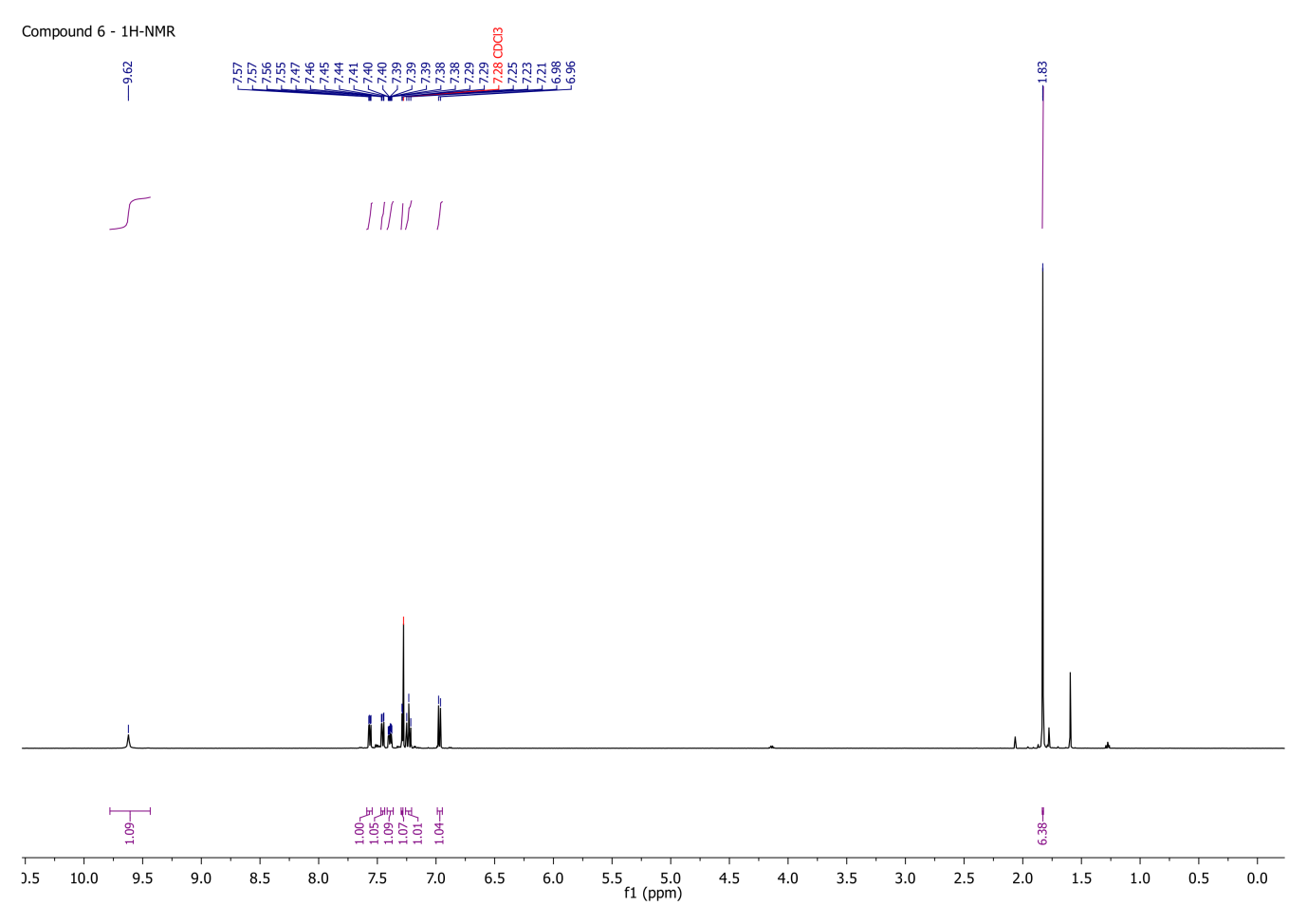

**Figure S16.** ^1^H-NMR of *6-(3-chloro-4-fluorophenyl)-4,4-dimethyl-1H-benzo[d][1,3]oxazine-2(4H)-thione (****6****).*


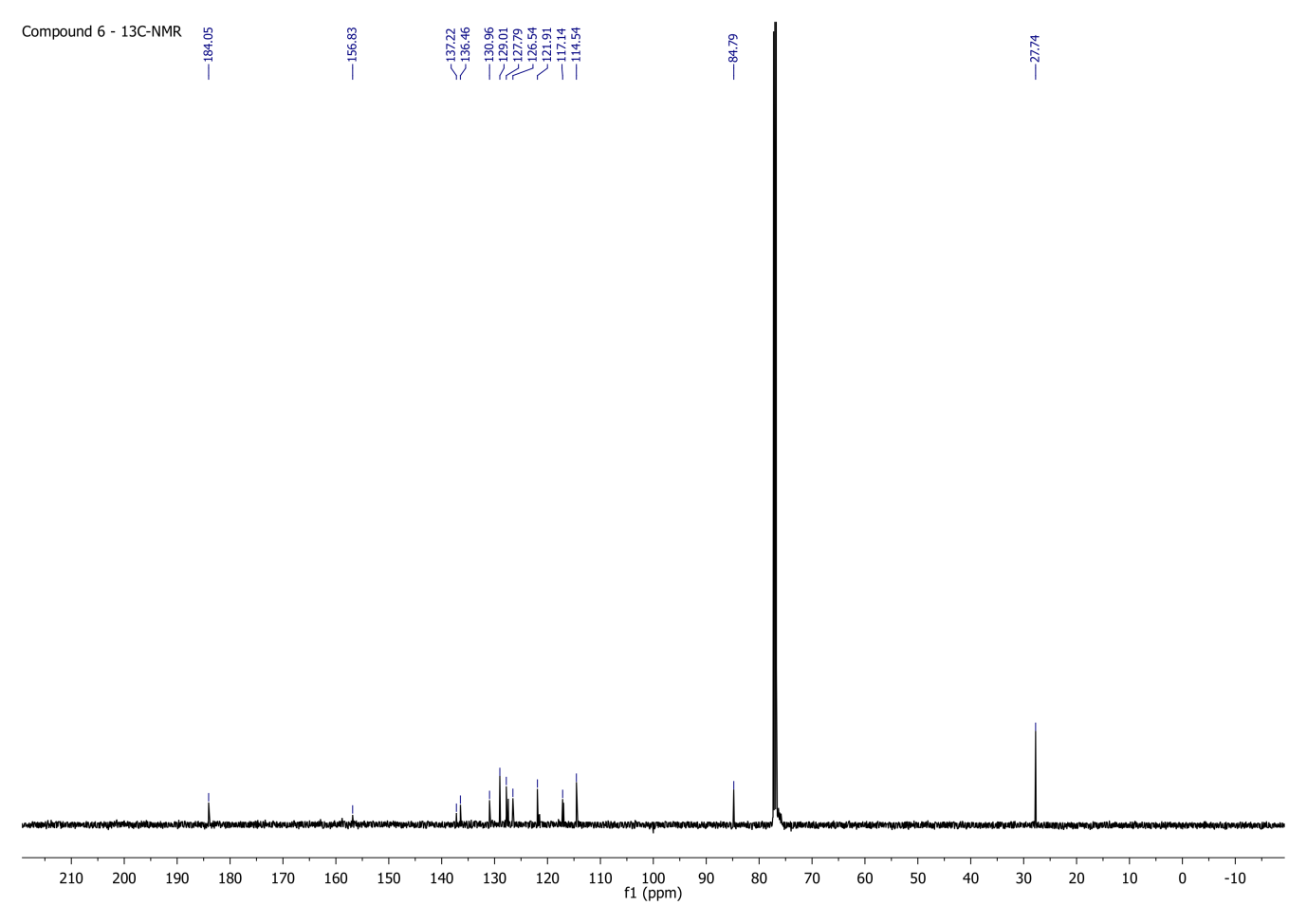

**Figure S17.** ^13^C-NMR of *6-(3-chloro-4-fluorophenyl)-4,4-dimethyl-1H-benzo[d][1,3]oxazine-2(4H)-thione (****6****).*


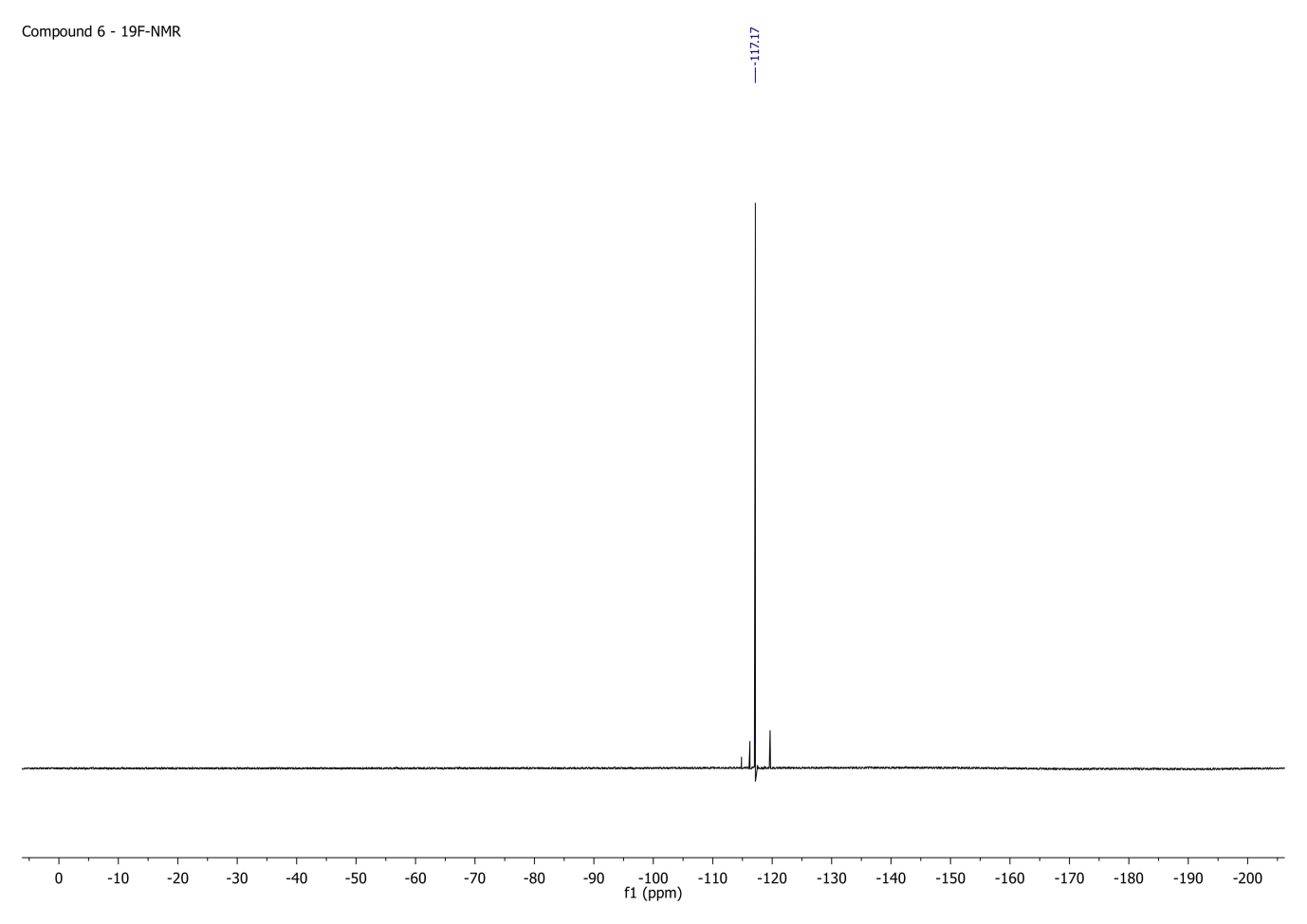

**Figure S18.** ^19^F-NMR of *6-(3-chloro-4-fluorophenyl)-4,4-dimethyl-1H-benzo[d][1,3]oxazine-2(4H)-thione (****6****).*

*
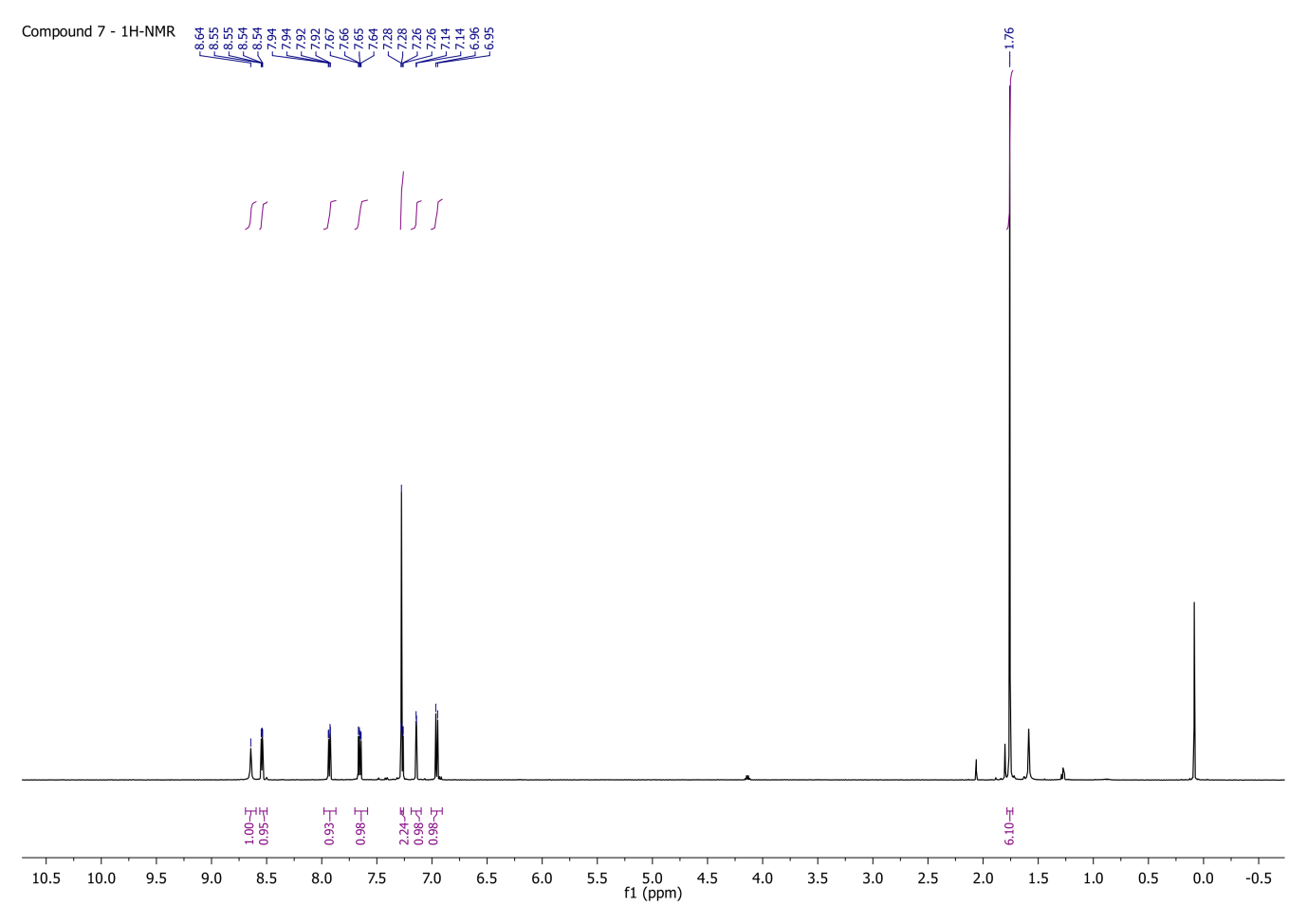
*

**Figure S19.** ^1^H-NMR of *4,4-dimethyl-6-(2-nitropyridin-3-yl)-1H-benzo[d][1,3]oxazin-2(4H)-one (****7****).*

*
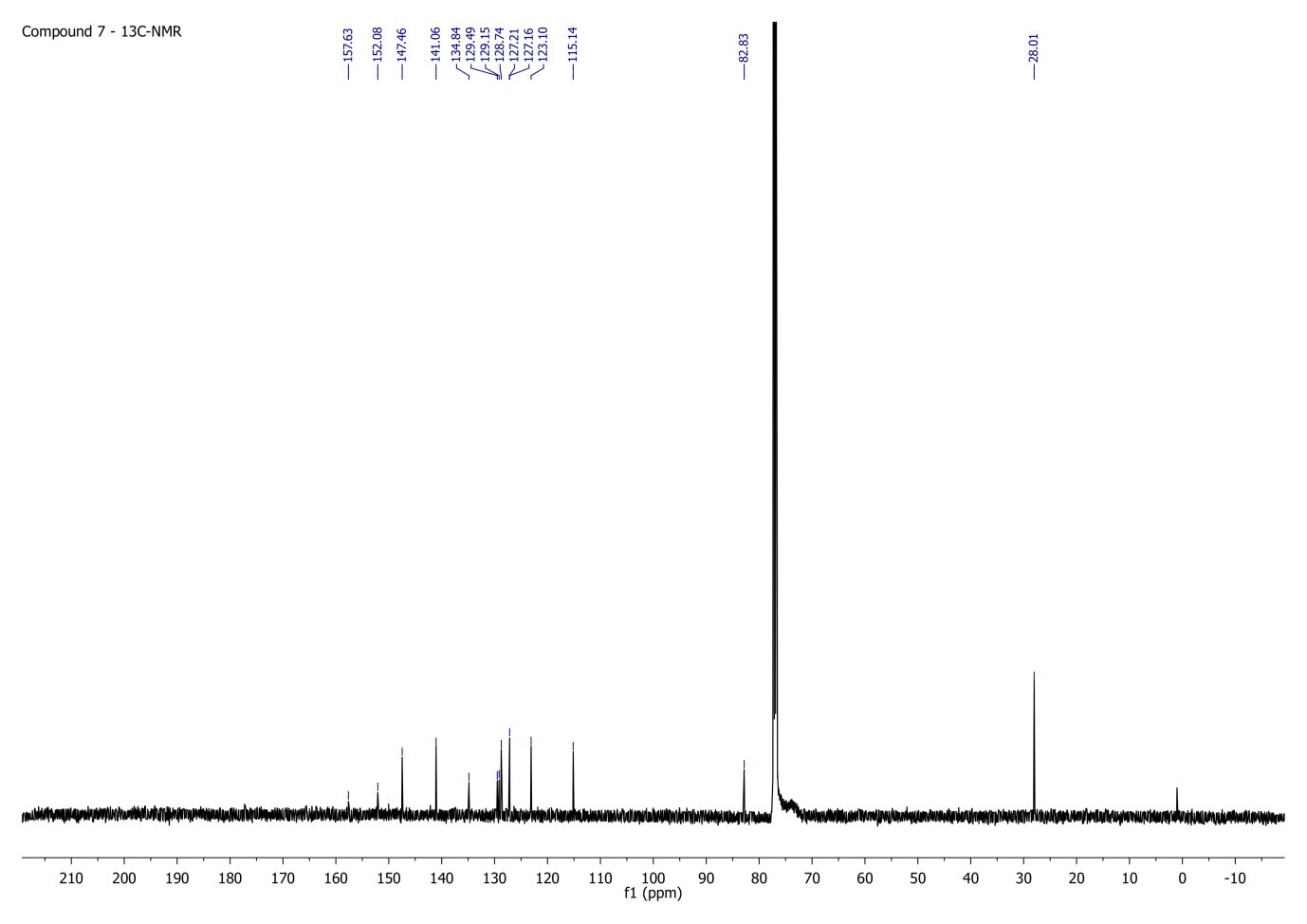
***Figure S20.** ^13^C-NMR of *4,4-dimethyl-6-(2-nitropyridin-3-yl)-1H-benzo[d][1,3]oxazin-2(4H)-one (****7****).*

**4. Purity RP-HPLC chromatograms**

**Figure S21**. RP-HPLC chromatogram of compound **1** (6-(2-fluoropyridin-3-yl)-4,4-dimethyl-1H-benzo[d][1,3]oxazin-2(4H)-one) using gradient 1.


**Figure S22**. RP-HPLC chromatogram of compound **2** (6-(2-fluoropyridin-3-yl)-4,4-dimethyl-1H-benzo[d][1,3]oxazine-2(4H)-thione) using gradient 1.

**Figure S23.** PR-HPLC chromatogram of compound **3** (6-(6-fluoropyridin-3-yl)-4,4-dimethyl-1H-benzo[d][1,3]oxazin-2(4H)-one) using gradient 1.

**Figure S24.** PR-HPLC chromatogram of compound **4** (6-(6-fluoropyridin-3-yl)-4,4-dimethyl-1H-benzo[d][1,3]oxazine-2(4H)-thione) using gradient 1.

**Figure S25**. PR-HPLC chromatogram of compound **5** (6-(3-chloro-4-fluorophenyl)-4,4-dimethyl-1H-benzo[d][1,3]oxazin-2(4H)-one) using gradient 1.

**Figure S26.** PR-HPLC chromatogram of compound **6** (6-(3-chloro-4-fluorophenyl)-4,4-dimethyl-1H-benzo[d][1,3]oxazine-2(4H)-thione) using gradient 1.

**Figure S27.** PR-HPLC chromatogram of compound **7** (4,4-dimethyl-6-(2-nitropyridin-3-yl)-1H-benzo[d][1,3]oxazin-2(4H)-one) using gradient 1.

**5. Radiochemistry RP-HPLC chromatograms**

Analytical and semi-preparative RP-HPLC were carried out on an Agilent Infinity 1200 system (Agilent Technologies). Elution profiles were analysed using Laura software (Lablogic, Sheffield, UK). Reaction progress and quality control checks were carried out with a Phenomenex Luna 5µ C18 100Å 150 × 4.60 mm column using gradient **2**: 0–25 min. 20%-80% B, 25-26min 80%-20% B, 26-35min 80%B with water as eluent A and methanol as eluent B. Semi-preparative RP-HPLC for the purification of **[^18^F]2** was performed with a Phenomenex Ultracarb 7µ ODS 250 × 10.60 mm column using gradient **3**: 0-35 min. 20%-80% B with water as eluent A and methanol as eluent B.

**Figure S28.** RP-HPLC chromatograms showing a representative example of [^18^F]fluoride incorporation into precursor **8** to synthesize **[^18^F]1** using gradient 2.

**Figure S29.** RP-HPLC chromatograms showing a representative example of conversion of **[^18^F]1** oxo-carbamate into **[^18^F]2** thio-carbamate using gradient 2.

**A**

**B**


**C**

**Figure S30.** RP-HPLC chromatograms showing a representative example of purified **[^18^F]2** using gradient 2; A) radio-channel with ≥95% RCP; B) UV-profile, region 1 and region 2 are suspected benzoxazin(thi)one containing compounds. C) UV-profile of compound **2** reference material spiked with **[^18^F]2** to confirm the identity of the product.

**6. Molar activity calibration curve**

**A**

**B**

**Figure S31.** A) Calibration curve used for calculating specific activity. B) Expanded portion of the the calibration curve. Data points expressed as the mean of triplicate determinations.

**7. Distribution coefficient analysis (LogD_7.4_)**

**Table S1.** LogD_7.4_ determination from three repeats (Run 1, Run 2, Run 3) with triplicate measurements (a, b, c) from each repeat.

|  | Octanol / PBS | LogD_7.5_ |
| --- | --- | --- |
| **Run 1 a** | 36.70 | 1.56 |
| **Run 1 b** | 69.79 | 1.84 |
| **Run 1 c** | 62.86 | 1.79 |
| **Run 2 a** | 57.79 | 1.76 |
| **Run 2 b** | 45.96 | 1.66 |
| **Run 2 c** | 49.06 | 1.69 |
| **Run 3 a** | 42.76 | 1.63 |
| **Run 3 b** | 47.19 | 1.67 |
| **Run 3 c** | 42.09 | 1.62 |
| **Mean ± SD (%)** | | **1.69 ± 0.1** |

**8. *In vitro* T47D AP assay**

|  |  |
| --- | --- |
| *^a^***Compound 1.** IC_50_ = 844.8 ± 0.23 nM | **Compound2.** EC_50_ = 4.7 ± 0.07 nM |
|  |  |
| *^a^***Compound 3.** IC_50_ = 795.0 ± 0.25 nM | **Compound 4.** EC_50_ = 3674.0 ± 0.08 nM |
| ***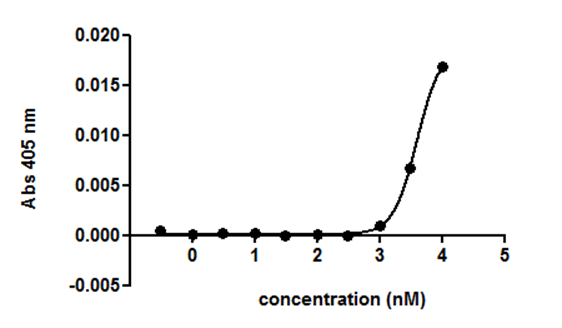*** |  |
| **Compound 5.** EC_50_ = ~4000 nM | **Compound 6.** EC_50_ = 432.5 ± 0.24 nM |

**Figure S32.** Plotted data from the T47D alkaline phosphatase potency assay. Data represented as the mean of at least n=3 determinations ± standard deviation. *^a^* IC_50_ _­_data calculated from the competition with progesterone (3 mM) because EC_50_ > 10,000 nM, indicating an antagonist biological profile.

**9. *In vitro* Cross-reactivity**

*

*

**Figure S33.** GR nuclear translocation assay (DiscoveRx^TM^) showed that compound **2** was a poor binder to GR. Dexamethasone was used as a positive control.

**10. In vivo evaluation**

**
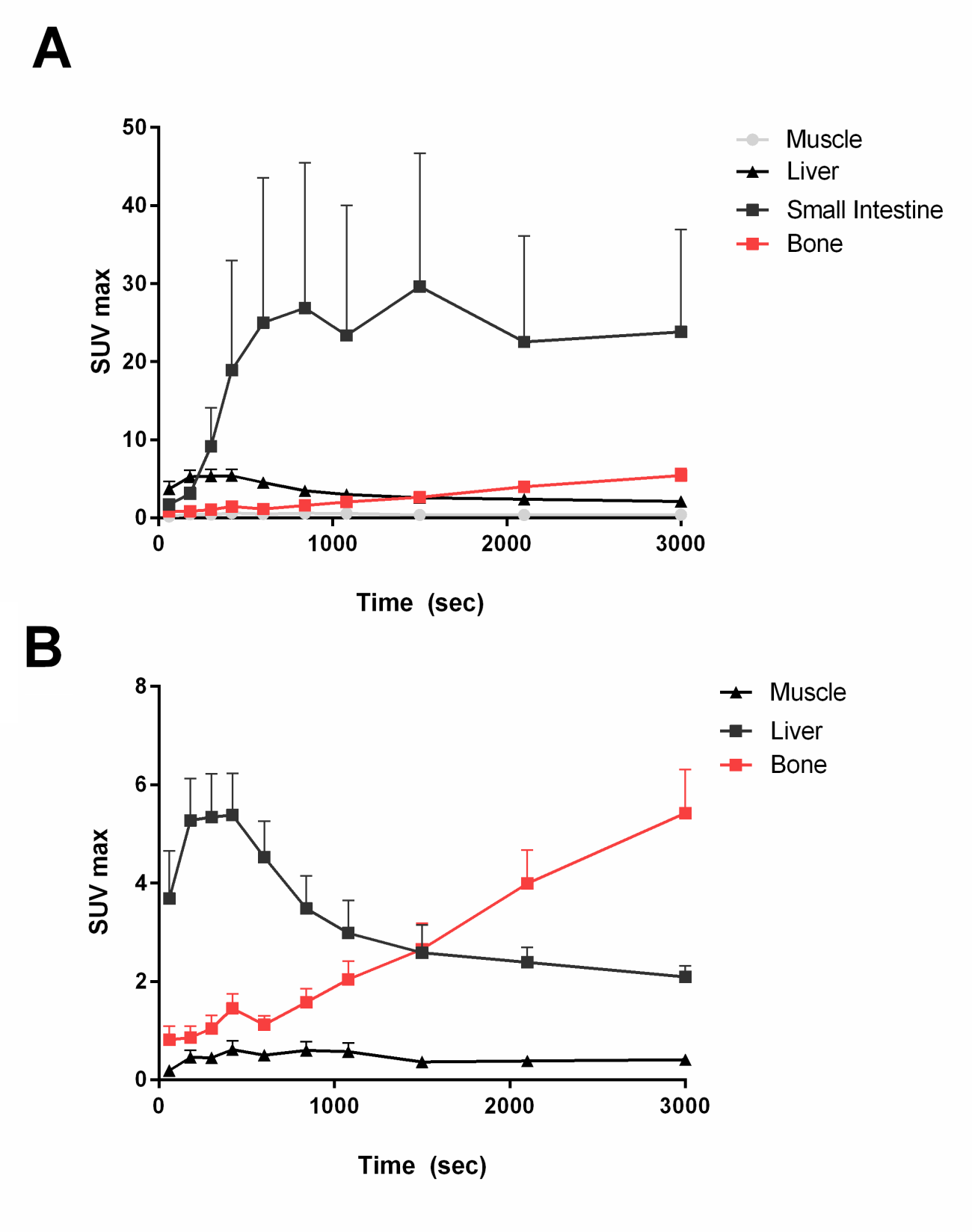
**

**Figure S34.** Time-activity curves (TAC) showing A) muscle, liver and bone, including small intestine B) muscle, liver and bone, excluding small intestine. TACs calculated as an average of the ROI count densities and data represented as mean ± SEM, (n = 3).

**Table S2.** Biodistribution results for **[^18^F]2** at 50 min p.i in control (PR-) and PR-induced (PR+) mice. The data are reported as the mean percentage of the injected dose per gram of tissue (%ID/g) ± SEM.

|  | Compound **[^18^F]2** | |
| --- | --- | --- |
| tissue | Control | PR-induced |
| blood | 0.01 ± 0.000 | 1.30 ± 0.30 |
| heart | 1.40 ± 0.33 | 1.47 ± 0.31 |
| lungs | 1.28 ± 0.35 | 1.33 ± 0.28 |
| bone | 15.37 ± 3.47 | 31.21 ± 5.99 |
| muscle | 0.91 ± 0.19 | 1.55 ± 0.44 |
| brain | 0.41 ± 0.09 | 0.40 ± 0.09 |
| kidney | 4.02 ± 1.18 | 3.89 ± 1.26 |
| liver | 6.19 ± 1.74 | 14.48 ± 4.91 |
| stomach | 0.73 ± 0.17 | 3.41 ± 0.26 |
| spleen | 0.99 ± 0.28 | 0.15 ± 0.06 |
| large intestine | 1.30 ± 0.07 | 13.25 ± 1.70 |
| small intestine | 30.49 ± 3.05 | 36.08 ± 1.21 |
| ovary | 1.32 ± 0.22 | 0.11 ± 0.03 |
| urine | 10.94 ± 8.40 | 12.93 ± 12.56 |

**10. *In vitro* metabolism of [^18^F]2**

10.1 Extraction efficiency

**Table S3.** Extraction efficiency data; protein pellet activity expressed as % total counts from n = 3 determinations.

|  | Protein pellet activity (% total counts) | Extraction efficiency (%) |
| --- | --- | --- |
| **Sample 1** | 6.8 | 93.2 |
| **Sample 2** | 7.8 | 92.2 |
| **Sample 3** | 4.8 | 95.2 |
| **Mean ± SD (%)** | | **93.5 ± 1.6** |

10.2 RP-HPLC chromatograms

Analytical RP-HPLC of radioactive metabolites was carried out using an Agilent Infinity 1200 system (Agilent Technologies). Elution profiles were analysed using Laura software (Lablogic, Sheffield, UK). The analytical RP-HPLC column, Phenomenex Luna 5µ C18 100Å 150 × 4.60 mm was used with gradient **3**: 15 min. 37% MeCN + 0.1%TFA / 63% Water + 0.1%TFA

**A**

**B**

**Figure S35.** Representative RP-HPLC chromatogram showing the metabolic conversion of **[^18^F]2** into **[^18^F]1** in MLM over 60 min; A) t = 0 min, B) t = 60 min.

**Table S4.** HPLC integration data for in vivo metabolite analysis of **[^18^F]2** using MLM from n = 3 determinations.

|  | **[^18^F]1 metabolite (R_t_ = ca 6:52 min:sec)** | **[^18^F]2 parent (R_t_ = ca 12:21 min:sec)** | **[^18^F] polar metabolites(R_t_ = ca 2:00 – 5:00 min:sec)** |
| --- | --- | --- | --- |
| **Run 1** | 62.3 % | 33.3 % | 4.4 % |
| **Run 2** | 59.3 % | 36.3 % | 4.4 % |
| **Run 3** | 59.0 % | 36.6 % | 4.4 % |
| **Mean ± SD (%)** | **60.2 ± 1.8 %** | **35.4 ± 1.8 %** | **4.4 ± 0 %** |

**A**

**B**

**Figure S36.** Representative RP-HPLC chromatograms showing the metabolic conversion of **[^18^F]2** into **[^18^F]1** in MLM over 60 min using parent radioligand **[^18^F]2** spiked with **[^18^F]1** to confirm the identity of the metabolite. A) Parent radioligand **[^18^F]2** spiked with **[^18^F]1** before incubation with MLM. B) RP-HPLC chromatogram of metabolites after 60 min incubation with MLM.

**11. Mass Spectrometry Metabolite Identification**

**Table S5.** Summary data from MLM metabolite identification of **[^18^F]2**.

**
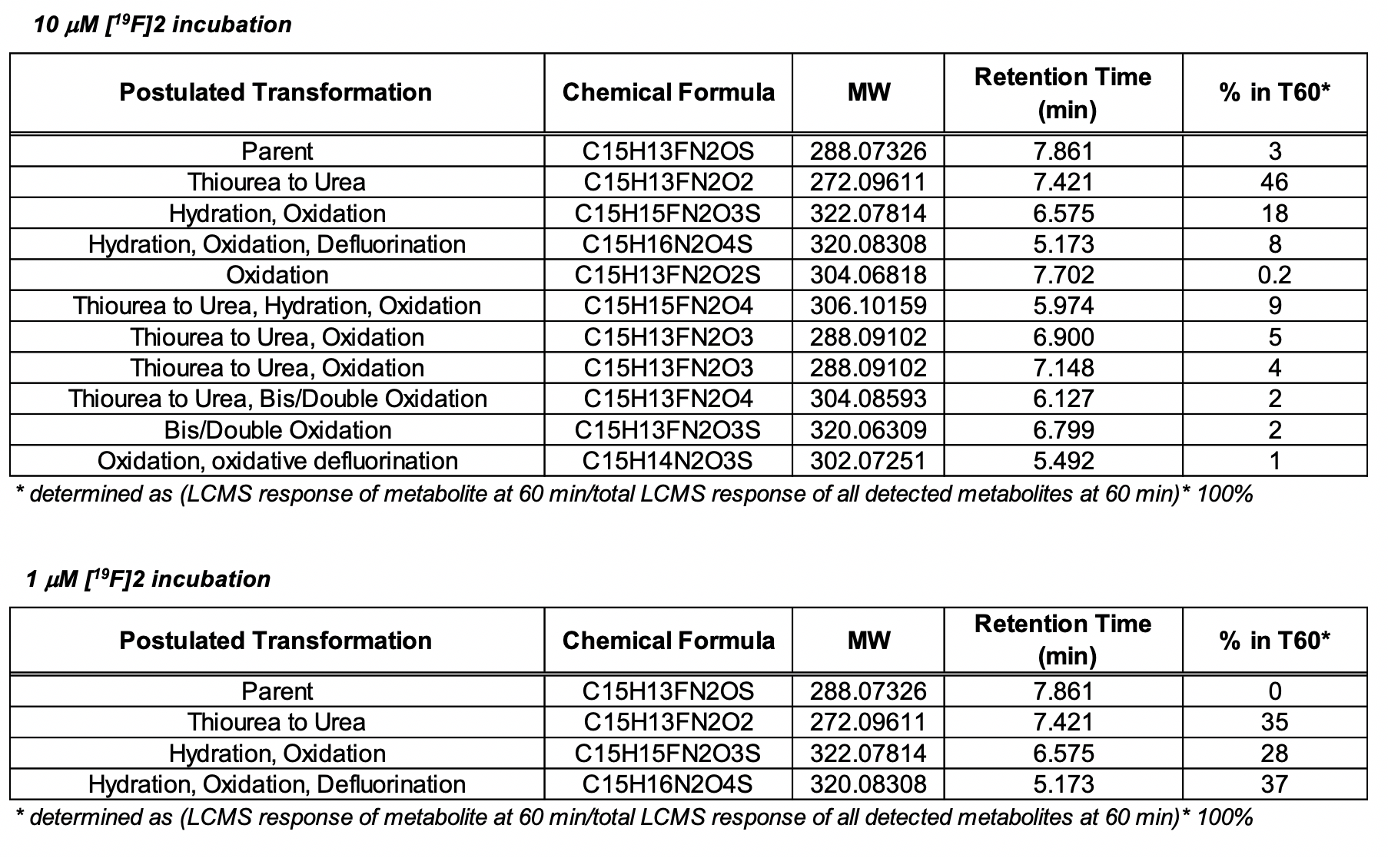
**

**Table S6.** Summary data from HLM metabolite identification of **[^18^F]2**.


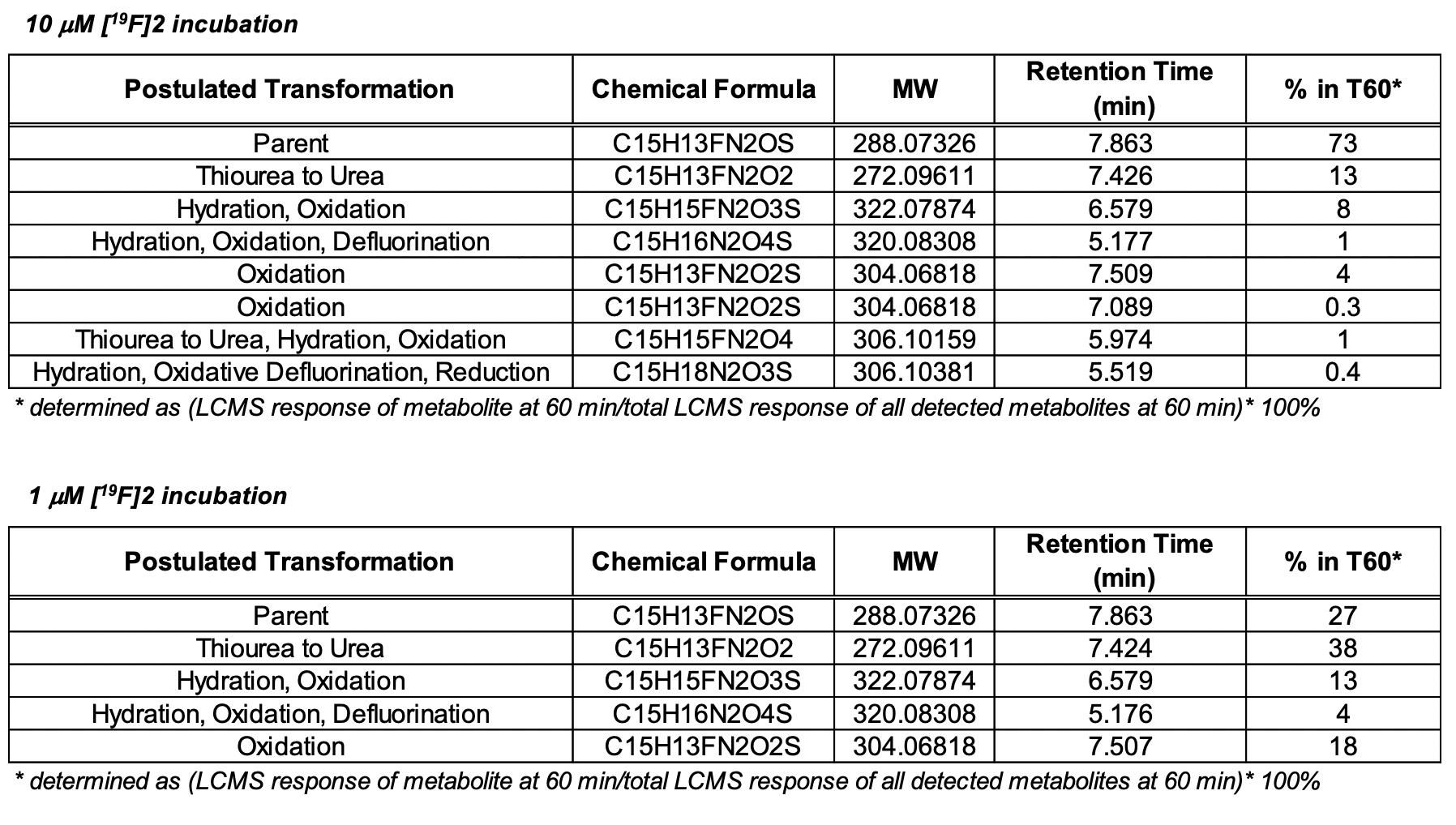

Supplement: Supplementary file 1 — Reaction Scheme for synthesis of precursor 7; 1H, 13C, 19F NMR data and HPLC purity analysis for compounds 1, 2, 3, 4, 5, 6 and 7; radio-HPLC analysis of [18F]1 and [18F]2; Molar activity calibration curve; Log D calculation raw data; plotted data for T47D assay; In vitro cross reactivity assay data; TACs for in vivo evaulation of [18F]2; ex vivo biodistribution data for [18F]2; HPLC metabolite analysis for [18/19F]2; MS metabolite analysis for [19F]2. (DOCX 3180 kb) [file 41181_2018_54_MOESM1_ESM.docx]
